# Who will import hydrogen in 2050? Global assessment with China and U.S. case studies

**DOI:** 10.1016/j.isci.2026.118175

**Published:** 2026-11-09

**Authors:** Veronika Brooks, Joshua Fragoso García, Lin Zheng, Viktor Paul Müller, Christoph Nolden, Dominik Möst, Martin Wietschel

**Affiliations:** 1 Fraunhofer Research Institution for Energy Infrastructures and Geotechnologies IEG, 03046 Cottbus, Germany; 2 Chair of Energy Economics, Technical University Dresden, 01069 Dresden, Germany; 3 Fraunhofer Institute for Systems and Innovation Research ISI, 76139 Karlsruhe, Germany

**Keywords:** hydrogen market, hydrogen imports, hydrogen demand, hydrogen supply, meta-analysis, renewable hydrogen, renewable energy potential, hydrogen trade

## Abstract

This study assesses the global hydrogen import requirements in 2050 by analyzing renewable hydrogen production potential in prospective import-oriented countries. Candidate importers are identified using GIS-based renewable energy potentials and projected 2050 primary energy demand. Two approaches are applied: (1) a meta-analysis of published hydrogen demand and production potential estimates, and (2) detailed regional analyses for the United States and China. The literature-based analysis indicates regionally concentrated imports of 128–1,255 TWh, with median and mean estimates of 458 and 438 TWh. Germany, Italy, and the Netherlands emerge as likely net importers, while several Asian countries have sufficient renewable resources to cover projected demand. The United States is well positioned to meet its demand domestically, whereas eastern China could require an additional 0–550 TWh of imports, depending on the share met internationally. Overall, import requirements depend on renewable resource distribution, energy demand, alternative hydrogen production pathways, and infrastructure constraints.

## Highlights

• Combined meta-analysis and case-study approach to hydrogen import requirements
• Global hydrogen import requirements in 2050 range from 128 to 1,255 TWh
• Hydrogen trade in 2050 is likely to remain largely regional
• U.S. demand can be met domestically, while eastern China may require imports

## Graphical abstract

**Figure:**
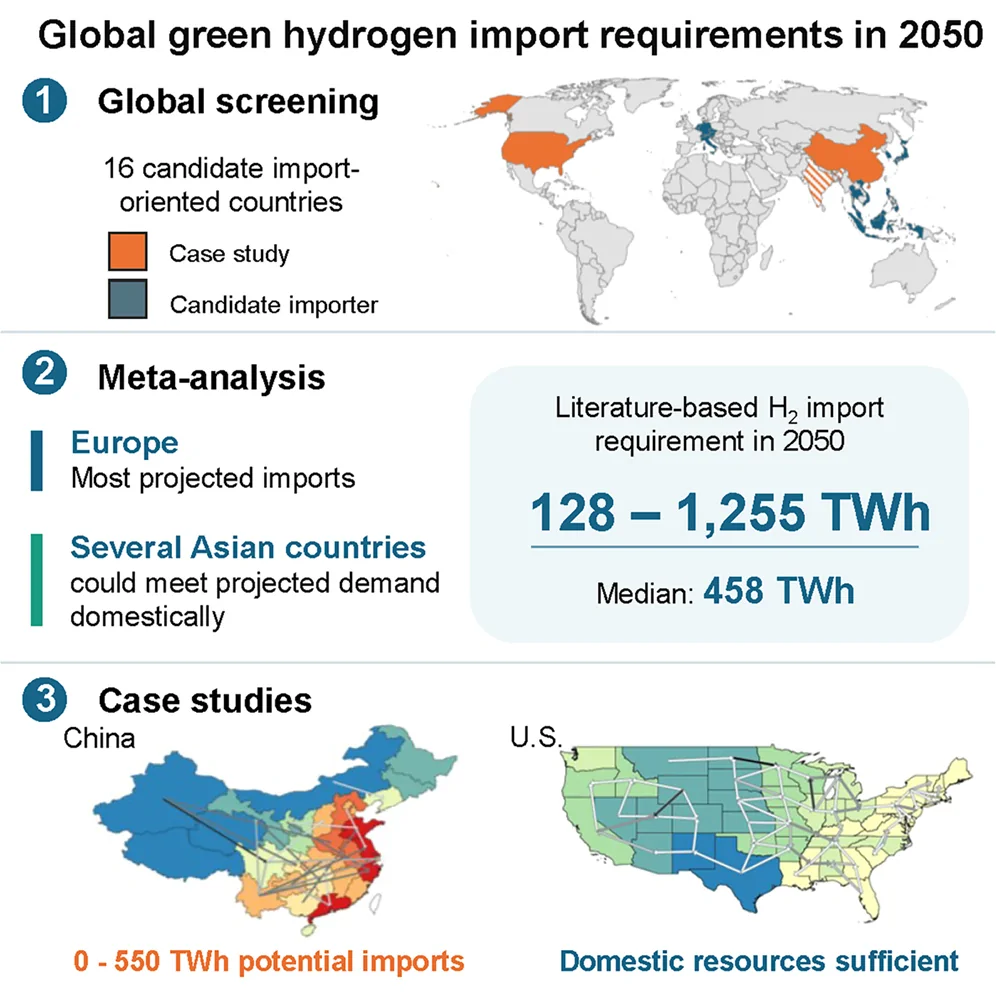

## Teaser

Applied sciences; Energy Resources

## Introduction

In recent years, green or sustainable hydrogen has gained prominence as a low-carbon energy carrier. The IPCC highlights its potential to connect sectors and decarbonize hard-to-abate industries.^1^ Its role is expanding beyond chemical feedstocks to include long-term energy storage, aviation and maritime transport, and high-temperature industrial processes.^2^ Forecasts suggest the global hydrogen market could reach USD 1.4–1.6 trillion by 2050,^3^ with demand projected between 4 and 15 PWh corresponding to equivalent of 4–11% of global energy use.^2^^,^^4^ Many countries have published hydrogen strategies that outline domestic demand and, in some cases, export ambitions.^5^ These evolving roles are shaped by geopolitical factors, the availability of renewable resources, and the economic context. Yet, future hydrogen trade patterns remain uncertain. Potential hydrogen trade will mainly be influenced by factors such as production costs, the types of products transported, and synthesis or conversion process steps. Additionally, producing low-carbon hydrogen domestically could be more economically attractive than importing it from countries with vast resources, given the losses incurred in the complex supply chain. Monitoring of announced projects on hydrogen supply revealed slower progress compared to a year ago.^6^ Despite these delays, recent studies suggest that hydrogen trade could emerge globally, prompting many countries to outline prospective supply chains.^7^^,^^8^^,^^9^^,^^10^

The Hydrogen Council and McKinsey identify key future hydrogen exporters by 2050, including the United States (U.S.), Canada, North African nations, Australia, the Middle East, and Latin America.^8^ They estimate that over half of global hydrogen demand could be met via long-distance transport of hydrogen or its derivatives. While some regions benefit from abundant low-cost renewables, countries such as the U.S. and Canada also rely on policy support, such as the U.S. Inflation Reduction Act and Investment Tax Credit.^8^

The IEA identifies potential hydrogen trade based on projects that have reached the final investment decision (FID) stage, including production, infrastructure, and potential offtakers. According to its Global Hydrogen Review 2026,^10^ the countries likely to be exporters by 2050 include Australia, the US, Latin America, Africa, the Middle East, and parts of Europe. These projections are based on announced export-oriented projects, although offtakers have been identified for less than a third of the planned capacity.^6^^,^^10^

Kim et al.^7^ highlight Australia, Saudi Arabia, Argentina, and Russia as potential hydrogen exporters based on their renewable energy potential. Global hydrogen atlases such as HYPAT, PtX Atlas, and EWI identify many more countries that could become hydrogen exporters.^11^^,^^12^^,^^13^ For example, Wietschel et al. 2024 (HYPAT)^11^ presents a global supply curve where 48 countries are selected to export hydrogen to Germany via various transport modes.

Several studies identify import-oriented regions, most notably countries such as Germany, the Netherlands, and Italy, as well as Japan, South Korea, and Singapore. These countries are characterized by limited domestic renewable energy resources and high industrial energy demand, making them likely candidates for hydrogen imports.^7^^,^^8^^,^^9^^,^^10^^,^^14^^,^^15^^,^^16^^,^^17^^,^^18^ Kim et al.^7^ claim that, apart from extensive hydrogen demand, overall reliance on energy imports, such as natural gas, coal, crude oil and its products, and electricity, as well as other factors including high electricity prices and land shortages, plays a role in favoring supply from other countries. One example is a potential future supply from Indonesia to South Korea. The study by Panchenko et al.^15^ encompasses a literature review on the global prospects of renewable hydrogen production, additionally defining China, South Africa, the U.S., and Chile as hydrogen importers from Australia. Hydrogen Council and McKinsey^8^ suggest that China, the United States, and India may meet most of their demand domestically but could still import from lower-cost producers such as the Middle East or Australia.

In the Global Hydrogen Review 2023,^17^ 2024,^9^ 2025^18^ and 2026,^10^ the IEA identifies the hydrogen-importing regions based on globally announced projects by matching the suppliers and offtakers. A significant share of projected hydrogen flows is assigned to undefined offtakers or projects without a FID. Export-oriented countries are clearly defined compared to the importing regions. This imbalance suggests an uncertainty in the global hydrogen trade, as there is a gap between supply readiness and a lack of clear demand centers.

Galimova et al.^19^ developed a global trade model to identify exporter and importer countries for synthetic fuels, such as e-methane, e-ammonia, and power-to-liquid (PtL), based on levelized costs. The study also assessed market value, trade volume, and geopolitical risks, with a focus on diversifying import portfolios. By 2050, exporters include regions in North and South America, sub-Saharan Africa, China, and Australia; importers are found in Europe, South and Southeast Asia, and parts of Africa. The model assumes fixed demand in 92 countries and does not account for domestic production potential in import-oriented countries. It also restricts each country to either an exporter or importer role, which oversimplifies the real-world dynamics, particularly for large countries such as the U.S. or China, where costs and demand vary regionally.

Many studies identify key hydrogen-exporting countries but the role of importers remains uncertain.^6^^,^^10^^,^^13^^,^^14^^,^^20^ While nations such as Germany, the Netherlands, Japan, and South Korea are expected to become major consumers, the broader global hydrogen import landscape is unclear.^9^^,^^10^^,^^15^^,^^17^^,^^18^^,^^19^^,^^21^^,^^22^^,^^23^^,^^24^^,^^25^ This study conducts a meta-analysis of national-level literature to estimate long-term renewable hydrogen import potential, focusing on hydrogen as the sole energy carrier. Countries are selected based on renewable resource availability and domestic energy priorities. The analysis includes 2050 projections of hydrogen production and demand. Additionally, two case studies on the U.S. and China are selected. A detailed analysis on the regional level is therefore conducted to assess their potential as importers, exporters, or both, which remains unclear in the current literature. This study answers the following research questions:

• Which countries could become the main importers of renewable hydrogen worldwide by 2050?
• What will be the size of the international hydrogen import requirements by 2050, given the hydrogen demand and potential of the identified countries?
• What are the energy policy implications of such developments?

To address these questions, we combine a global screening of domestic renewable resource availability with a literature-based meta-analysis of hydrogen demand and supply potential. This is complemented by subnational case studies for China and the United States to assess spatial differences between renewable hydrogen resources and demand. An overview of the analytical framework is provided in Figure 1, with the detailed methodology described in the Method Details section.

**Figure 1:**
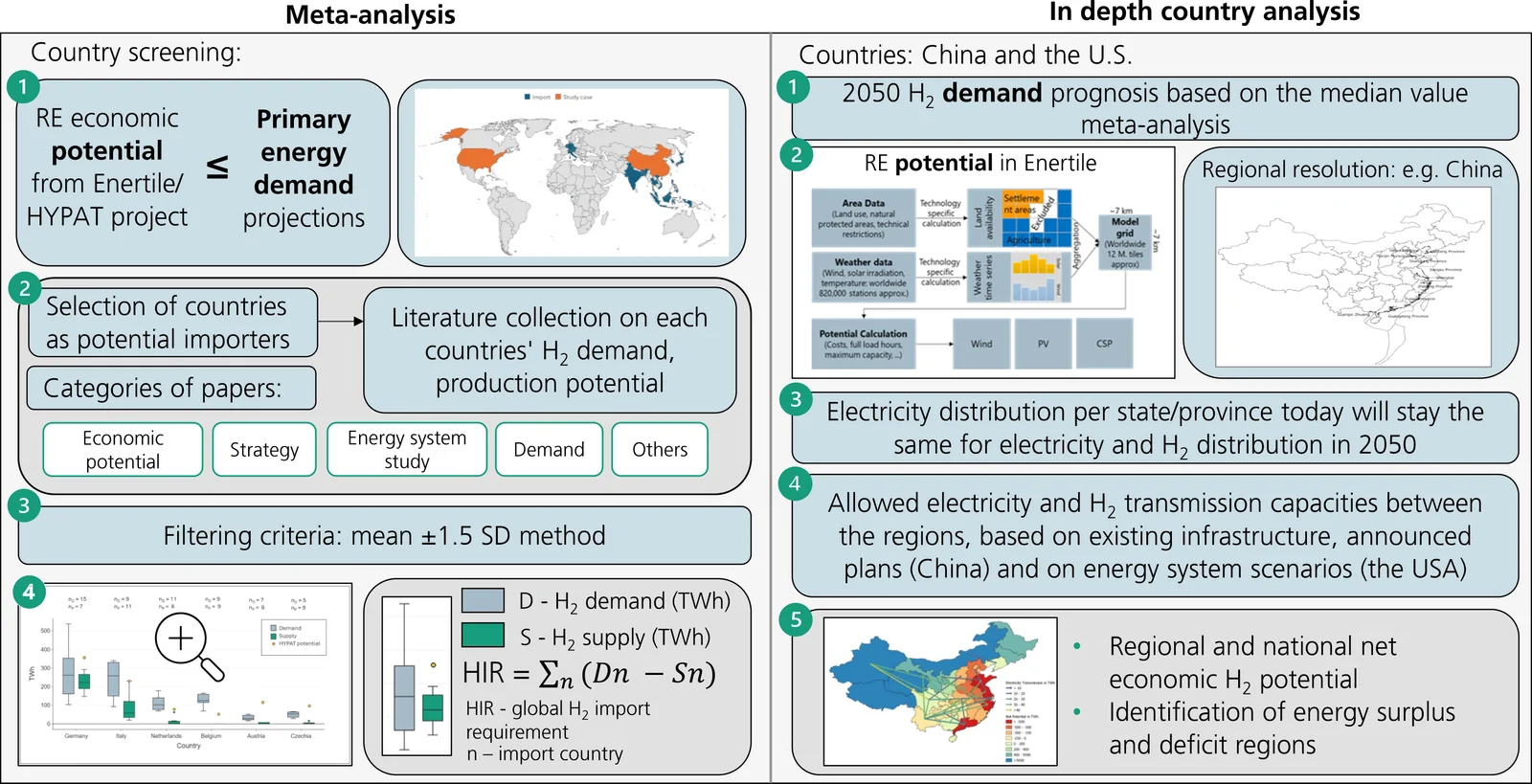
Overview of the analytical framework The left image shows the global country screening and literature-based meta-analysis, while the right image shows the subnational analysis applied to China and the United States. Detailed methods are provided in STAR Methods.

## Results

The initial global screening identified 16 countries with limited domestic renewable resource availability relative to projected energy demand (Table 1). These countries are concentrated mainly in Central Europe and East and Southeast Asia (Figure 2). The screening was conducted to set a basis for the literature-based comparison of hydrogen demand and domestic supply potential presented later in discussion. In addition to the selected countries, we conducted a more detailed analysis of China and the United States. These two countries were chosen because, although both have sufficient electricity generation potential to meet their demand by 2050, the locations of these resources do not necessarily align with the main demand centers. Furthermore, China and the United States are the world’s second- and third-largest countries by area, the first and second largest economies and the two biggest CO2 emitters.^26^^,^^27^^,^^28^ The case studies therefore assess hydrogen demand and renewable resource availability at the state/provincial level and complement the country-level meta-analysis. Figure 2 illustrates the countries selected for this study. Countries shown in blue indicate potential hydrogen importers, primarily located in two regions: Central Europe and Southeast Asia. The United States and China are marked in orange and are included as case studies. India was identified as a case study but was excluded from the paper's scope.

**Table 1:** Country selection based on a comparison of renewable electricity generation potential at 100 €/MWh with projected energy demand in 2050

Country	Sum of RE generation potential (TWh)	Primary energy demand (TWh)	Resource gap (TWh)	Considered in this study (shortfall larger than 30 TWh)
Slovenia	41	42	1	no
Malta	1	5	4	no
Bhutan	16	23	7	no
Bangladesh	431	448	17	no
Equatorial Guinea	35	52	17	no
Brunei	3	21	18	no
Luxembourg	12	44	32	yes
Czechia	205	242	37	yes
Netherlands	473	517	44	yes
Philippines	492	541	49	yes
Switzerland	92	183	91	yes
Bahrain	12	108	96	no
Trinidad and Tobago	20	129	109	no
Austria	144	259	115	yes
Singapore	0	248	248	yes
Belgium	122	395	273	yes
Vietnam	820	1196	376	yes
Italy	536	937	401	yes
Germany	1488	1952	464	yes
Thailand	971	1593	622	yes
Malaysia	371	1049	678	yes
India	8109	14774	6665	no
South Korea	324	2262	1938	yes
Japan	594	2928	2334	yes
Indonesia	994	4615	3621	yes

See Figure S1 and Table S1 for sensitivity analyses.

**Figure 2:**
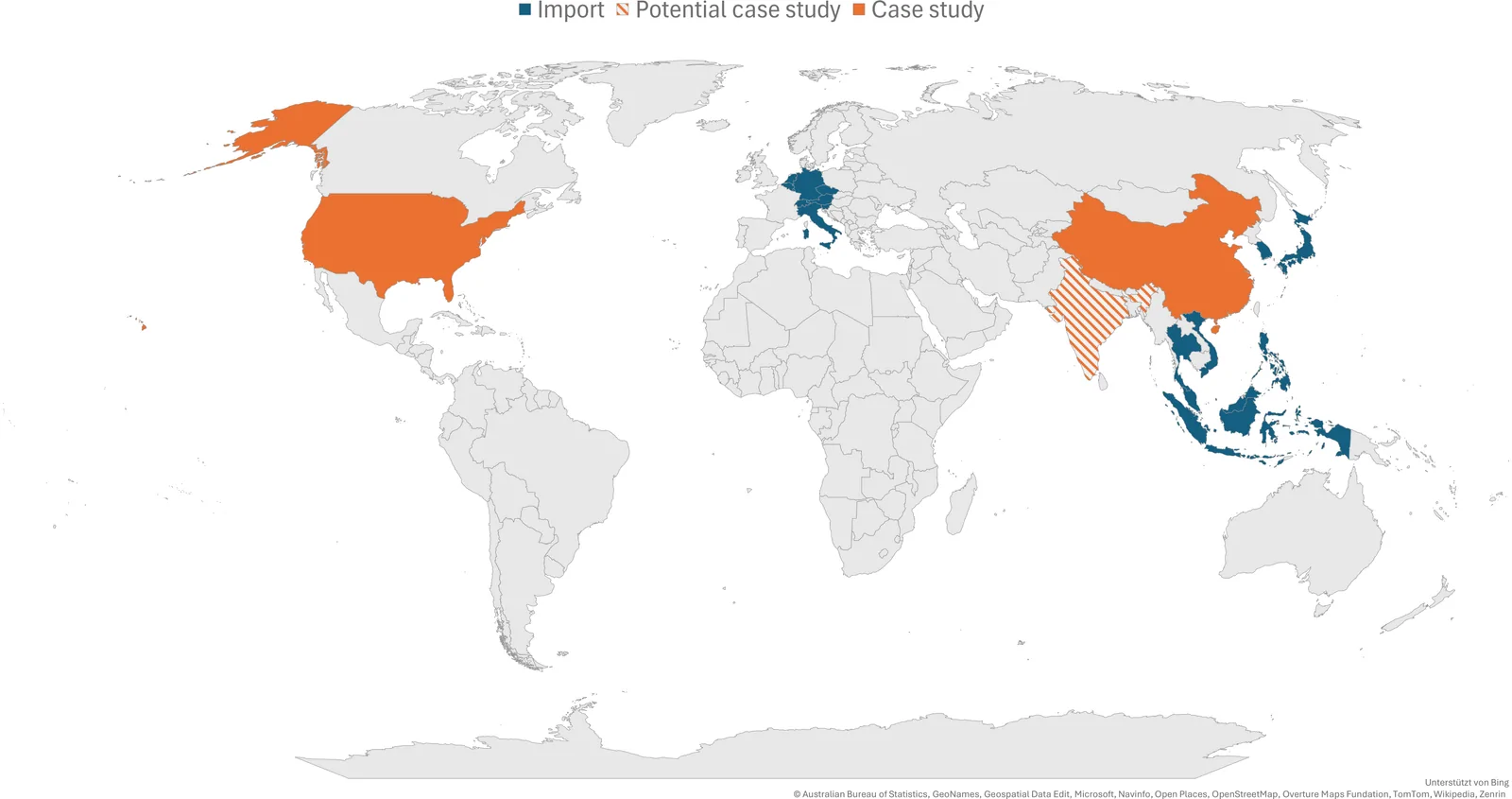
Geographical location of the countries selected for this study

### Regional meta-analysis

#### Hydrogen-importing countries of the European region

The meta-analysis of the European region focuses on Germany, Italy, the Netherlands, Belgium, Austria and the Czech Republic. They all exhibit the largest renewable energy deficits, mainly due to high primary energy consumption and insufficient land availability, resulting in limited low-cost renewable potential. Figure 3 summarizes the results of the meta-analysis on the hydrogen demand projections and the renewable hydrogen production potential. Subsequently, the number of values for each category (supply or potential) for each country should be at least five to analyze the boxplots. Countries that do not meet this criterion are excluded from the analysis and listed in Table S5. Figures 3 and 4 include the number of values considered for each box.

**Figure 3:**
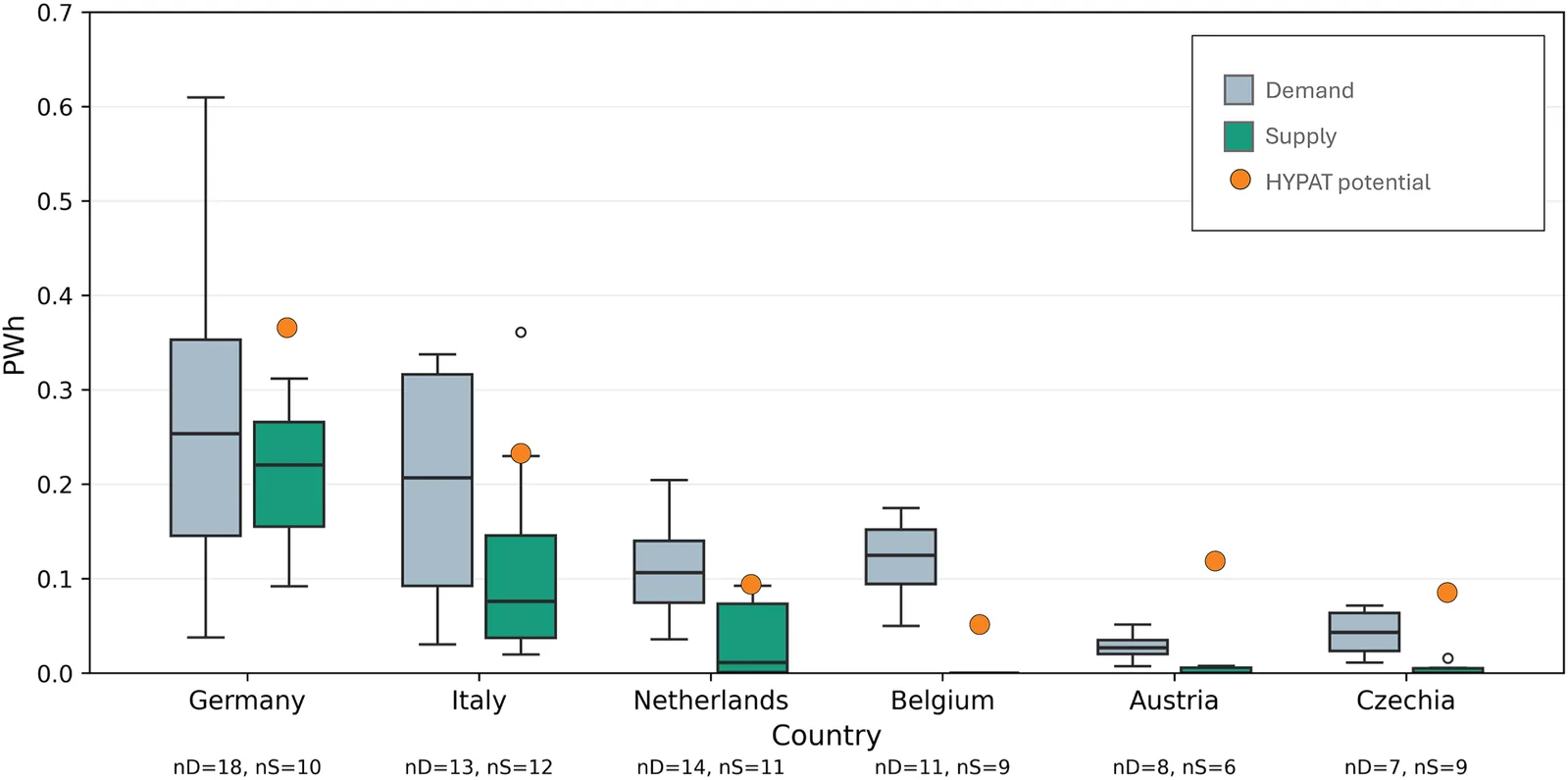
Literature overview of hydrogen supply potential and demand in selected European countries in 2050, with filtering based on the 1.5 SD limit Unfiltered results and results using a ±1 SD filter are presented in Figure S2. n_D_ and n_S_ denote the number of sample values for demand and supply potential, respectively, after filtering.^13^^,^^29^^,^^30^^,^^31^^,^^32^^,^^33^^,^^34^^,^^35^^,^^36^^,^^37^^,^^38^^,^^39^^,^^40^^,^^41^^,^^42^^,^^43^^,^^44^

**Figure 4:**
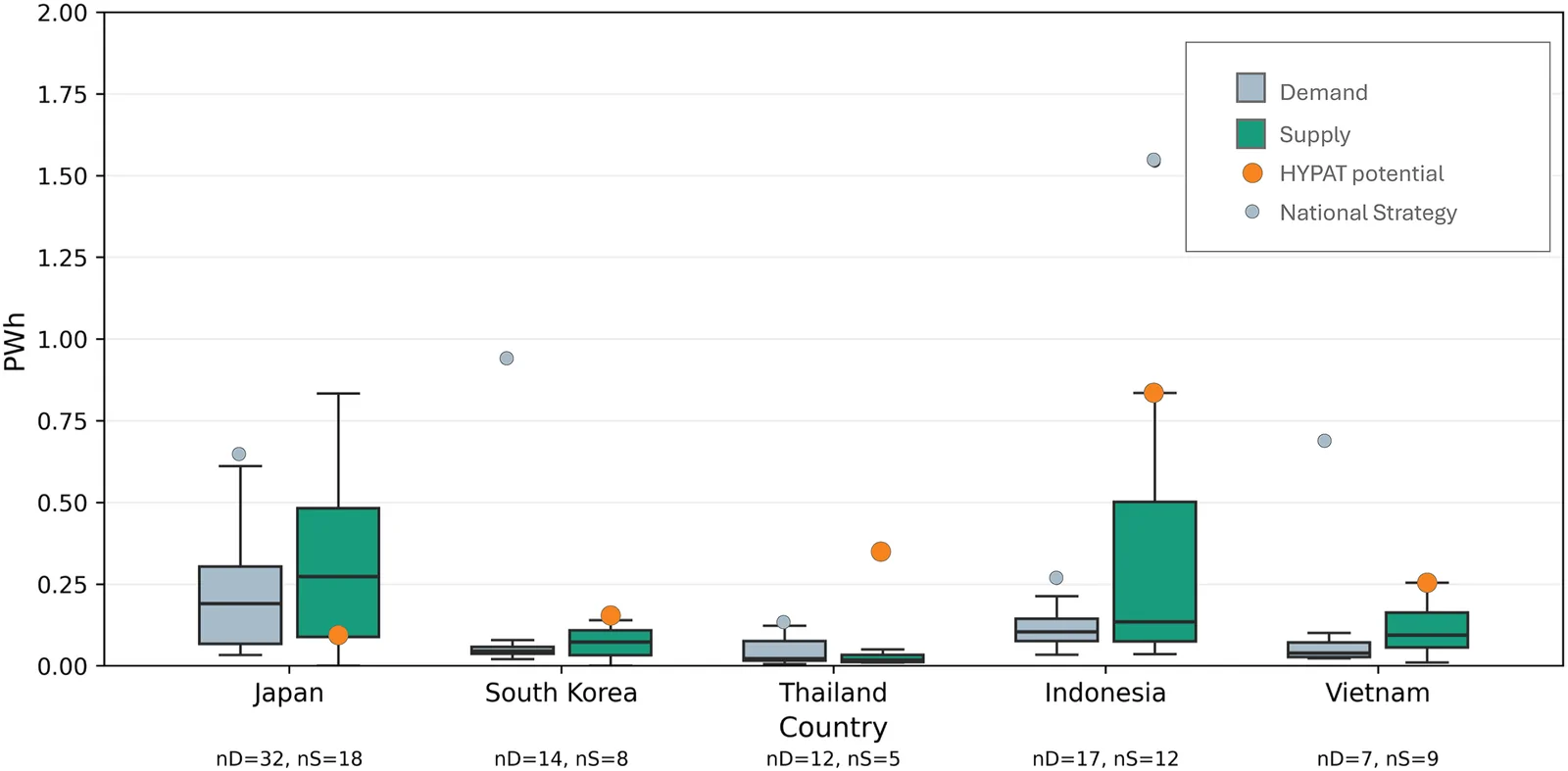
Literature overview of hydrogen demand and supply potential in selected Asian countries in 2050, with filtering based on the 1.5 SD limit Unfiltered results and results using a ±1 SD filter are presented in Figure S3. n_D_ and n_S_ denote the number of sample values for demand and supply potential, respectively, after filtering.^21^^,^^25^^,^^45^^,^^46^^,^^47^^,^^48^^,^^49^^,^^50^^,^^51^^,^^52^^,^^53^^,^^54^^,^^55^^,^^56^^,^^57^^,^^58^^,^^59^^,^^60^^,^^61^^,^^62^^,^^63^^,^^64^

Among the countries analyzed, the future projections in Germany and Italy show the highest demand and supply potentials with the largest spread, especially the hydrogen demand projections. This underlines the large cross-study spread of hydrogen demand in these two countries. In Germany, projected hydrogen demand spans from 40 to 610 TWh, while the domestic renewable hydrogen production potential ranges between 90 and 310 TWh. For Italy, hydrogen demand is estimated to range from 30 to 340 TWh, whereas the production potential remains more constrained, predominantly between 20 and 230 TWh. The production potential for the Netherlands, Belgium, Austria and Czech Republic does not exceed 15–20 TWh, due to their limited economic RE availability and the dominance of studies with system analysis.

Additionally, the orange markers in Figures 3 and 4 represent the economic potential for hydrogen production obtained from the HYPAT project. The economic threshold of 3 €/kg (100 €/MWh) was used to quantify the amount of hydrogen produced domestically, which aligns with projected hydrogen production costs reported by Bühler et al.^65^ The levelized cost of hydrogen (LCOH) methodology was developed and described by Mendler et al.^29^ For Austria and the Czech Republic, based on the HYPAT projections, the demand should be able to be covered domestically. However, the meta-analysis, based on the literature with a high share of energy system studies, indicates importing hydrogen is more economical to cover the demand (Tables S2 and S3).

#### Hydrogen-importing countries of the Asian Region

The meta-analysis of the Asian region shows that the import potential is less distinct than the clearly defined patterns observed in Europe. Countries such as Japan, South Korea, Thailand, Indonesia, and Vietnam, see Figure 4, were selected for their significant deficit between renewable energy potential and primary energy consumption. The results do not necessarily show the high hydrogen import potential as some studies concluded in the literature review section. After applying the 1.5 SD limit, only Thailand showed higher hydrogen demand than renewable hydrogen production potential. Based on the detailed literature review, countries such as Japan, South Korea, and Indonesia could theoretically decarbonize their hydrogen demand through domestic renewable energy resources, as well as their high reliance on nuclear energy. Nevertheless, the economic feasibility of these resources remains a critical factor. For instance, while Japan possesses significant offshore wind potential, much of it is in remote areas, therefore making it less economically attractive.^66^^,^^67^ Countries such as Singapore and the Philippines are excluded from the analysis due to an insufficient number of data points (fewer than five).

In the Asian region, national hydrogen strategies, and roadmaps, for example in Japan, South Korea, and Vietnam, report substantially higher hydrogen demand than many literature estimates, which often reflect demand in cost-optimal energy systems (Figure 4 and Tables S2 and S3). This indicates the ambitions of these countries to establish a national hydrogen economy or play a significant role in the international hydrogen trade. If such values are taken as a signal to international hydrogen suppliers, then import requirements will increase strongly. Additionally, a substantial share of the demand estimates for Japan, South Korea and Indonesia is derived from JRC projections.^68^ These values represent hydrogen demand within total final energy consumption and therefore exclude non-energy uses of hydrogen, such as feedstock for ammonia production, while hydrogen used in steelmaking is included in industrial energy demand.^68^ To assess the effect of this system boundary, we increased all JRC-derived hydrogen demand estimates by 10% to account for excluded non-energy feedstock uses; further details are in Methods S1. The median, mean, and low-import cases remain unchanged, while the high-import case increases by 6 TWh or 0.46%, from 1,255 to 1,261 TWh (Table S7). The literature dataset also includes national strategies and studies with broader sectoral coverage and higher projected demand, contributing to the wide range of reported hydrogen demand estimates.

The hydrogen production potential from the HYPAT project (orange markers in Figure 4) represents the economic potential for hydrogen production below 3 €/kg (100 €/MWh). In Japan, the renewable energy potential could reach 800 TWh, which could cover the demand by unlocking significant offshore wind resources. Due to the great depth of the sea, floating turbines could be an option, although this would raise the cost significantly.^67^ For Thailand and Indonesia, the economic hydrogen production potential is estimated at 335 TWh and 835 TWh, respectively, which could, according to selected studies, cover the demand.

### Focus countries

The literature review includes detailed analyses for the United States and China. Although both countries are expected to account for a substantial share of global hydrogen demand, they also possess significant net production potential and, at first glance, do not appear to be prospective importers (Figure 5). However, due to their large geographical size, it is important to assess renewable energy availability at a higher spatial resolution to identify optimal production locations and major demand centers. The findings from these two case studies are presented later in discussion.

**Figure 5:**
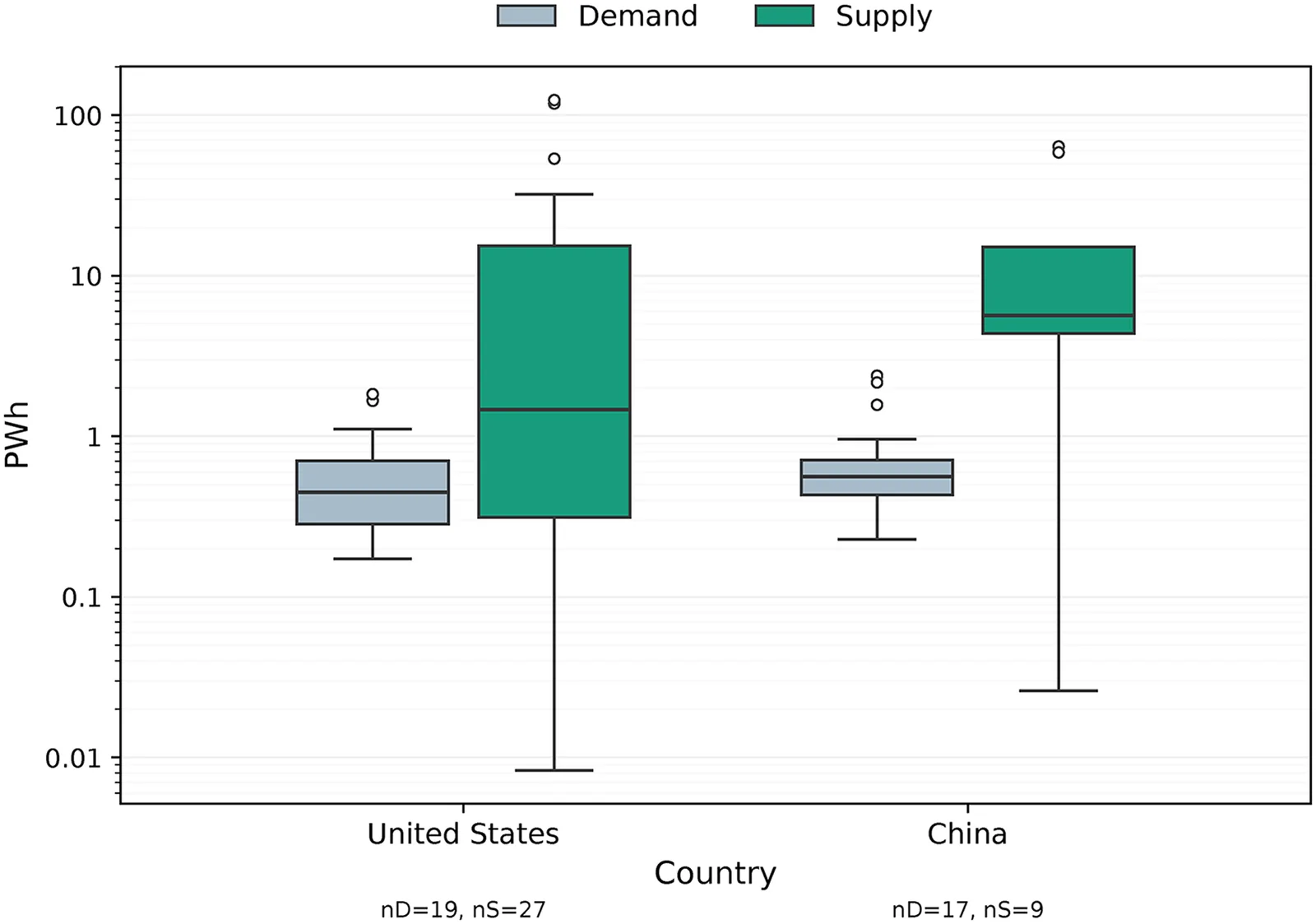
Literature overview on hydrogen supply potential in comparison to hydrogen demand in the U.S. and China, 2050 All collected values^12^^,^^13^^,^^14^^,^^29^^,^^32^^,^^46^^,^^47^^,^^48^^,^^49^^,^^68^^,^^69^^,^^70^^,^^71^^,^^72^^,^^73^^,^^74^^,^^75^^,^^76^^,^^77^ are shown without SD filtering to illustrate the range and scale of national-level demand and supply estimates. The numbers below each country show the number of collected demand (nD) and supply (nS) values. The y axis is logarithmic.

#### China case study

Figure 6 illustrates the geographical net potential of renewable energy resources in various Chinese provinces, representing the difference between the total available renewable energy and the total electricity and hydrogen demand in each province. The net potential includes the effect of the planned electricity transmission lines and hydrogen pipelines. Hydrogen is converted into an electrical equivalent. Hydrogen demand, when converted into electricity, accounts for 5.4% of the total demand.

**Figure 6:**
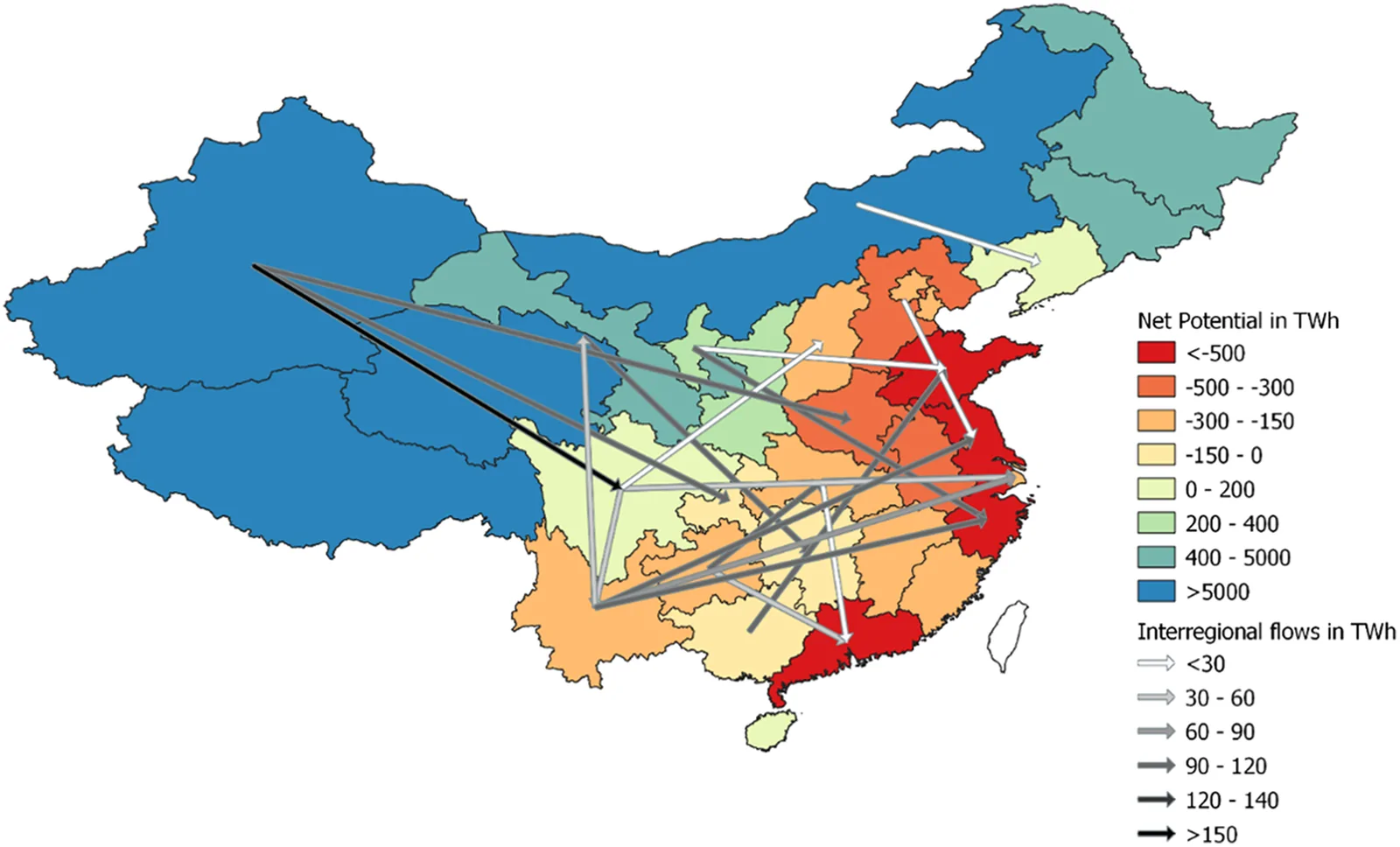
Net potential in Chinese regions (TWh) This accounts for the generation and demand of electricity and hydrogen (converted into electricity equivalents), as well as cross-regional imports and exports. The color scale shows regional net potential, while the arrows -shown in grayscale-represent the magnitude of the combined electricity and hydrogen flows between regions.

Most of the southeastern provinces, where many demand centers are located, have a significant deficit in renewable energy availability. The regions with the largest deficits include Shandong, Zhejiang, and Guangdong, each with a deficit exceeding 500 TWh. In addition, Hebei, Henan, Anhui, and Fujian each have a deficit of between 300 and 500 TWh. The central part of China has a balance between potential and demand, according to our projections.

Conversely, the northwestern provinces of Xinjiang, Tibet, Qinghai, Gansu, and Inner Mongolia have a high surplus, with each of these regions having a renewable energy potential of over 5,000 TWh. Although the planned transmission and gas infrastructure is designed to transfer energy from these resource-rich western provinces to the deficit-prone eastern regions, the overall shortage in the east remains a significant challenge.

Figure 7 illustrates the potential for renewable electricity generation by levelized cost of electricity (LCOE) tier, alongside provincial electricity demand across China. The electricity demand includes the additional energy required for hydrogen production within each province, further influencing the regional energy balance. Five regions, Xinjiang, Inner Mongolia, Tibet, Qinghai, and Gansu, stand out due to their exceptionally high surplus in renewable generation potential. Each of these provinces has the capability to generate over 3,500 TWh at an LCOE below 20 €/MWh. Their vast renewable resources position them as potential energy hubs, capable of supplying not only local demand but also energy-deficient regions through transmission networks. This is also underlined by the number and the scale of existing and planned transmission lines and pipelines. However, to cover the deficit in energy-deficient regions, more large-scale transmission lines and pipelines will be required.

**Figure 7:**
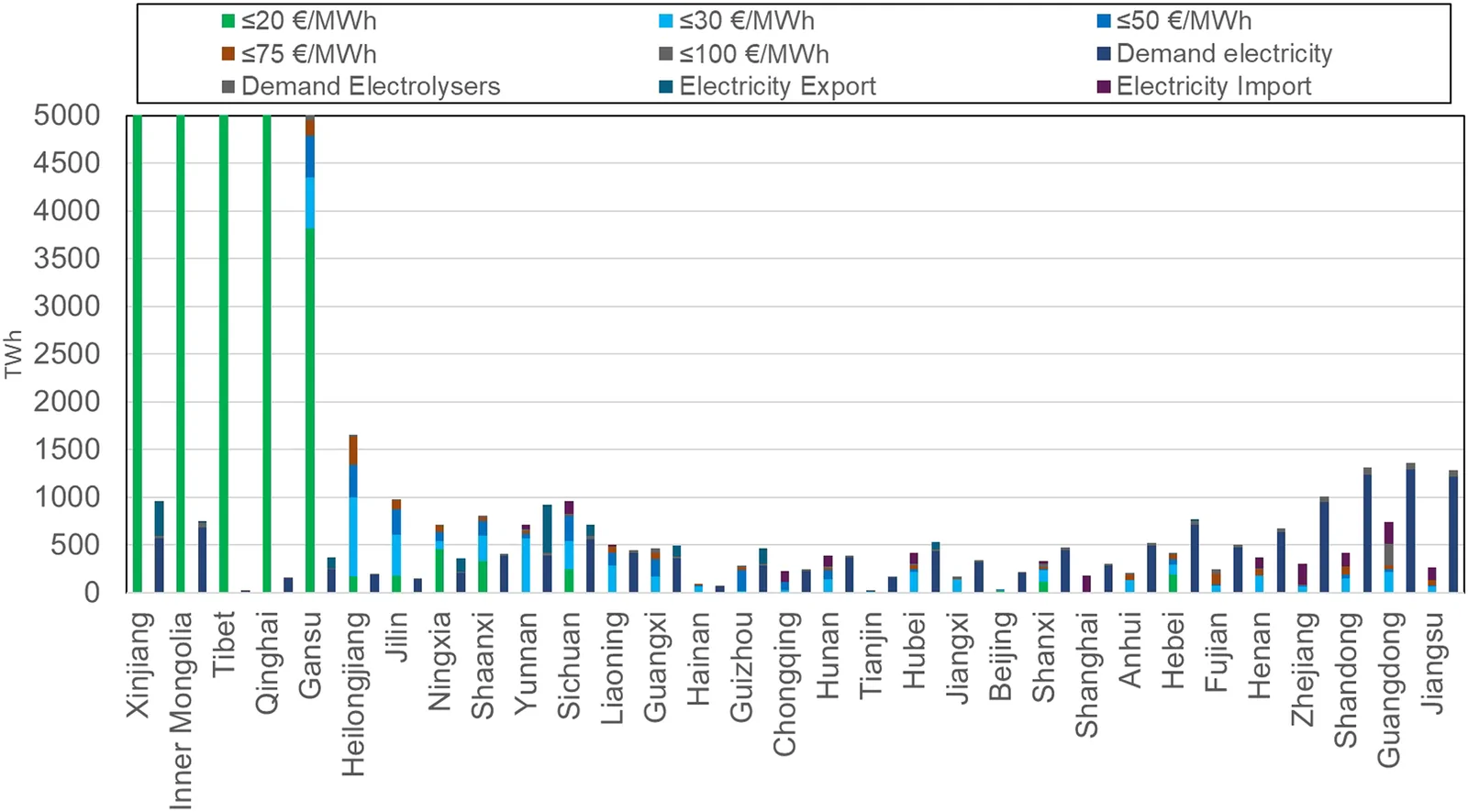
The potential for renewable electricity generation and the projected demand for electricity in China in 2050, broken down by province Generation potential is shown by the levelized cost of electricity (LCOE) ranges. Demand values include hydrogen demand converted into electricity equivalents. Inter-regional imports and exports are included in the regional net balance.

On the other hand, 14 provinces face a persistent generation deficit, even when considering higher-cost renewable generation options with LCOEs of up to 100 €/MWh. This highlights the challenges of balancing supply and demand across the country, as renewable resources and demand centers are unevenly distributed and the (planned) transmission capacity between them is still far behind what is needed. As previously noted in Figure 6, the provinces with the highest deficits include Shandong, Zhejiang, Guangdong, Hebei, Henan, Anhui, and Fujian. These regions will continue to rely on energy imports despite planned transmission networks, underscoring the need for further infrastructure development and energy diversification strategies. Moreover, the future energy demand in certain coastal provinces may be covered not only by other provinces in China but also by other countries through ships, as is currently the case for coal and liquefied natural gas (LNG).

#### U.S. case study

Figure 8 illustrates the geographical net potential of renewable energy resources across different U.S. states. Hydrogen demand, when expressed in terms of electricity, accounts for approximately 6.7% of the total electricity demand in 2050. All U.S. states exhibit a net surplus of renewable energy potential; no state faces an energy deficit. However, there are notable regional disparities in the available net potential. States along the East Coast have the lowest potential, ranging between 0 and 200 TWh. In contrast, Texas and New Mexico each have net potentials exceeding 2,000 TWh.

**Figure 8:**
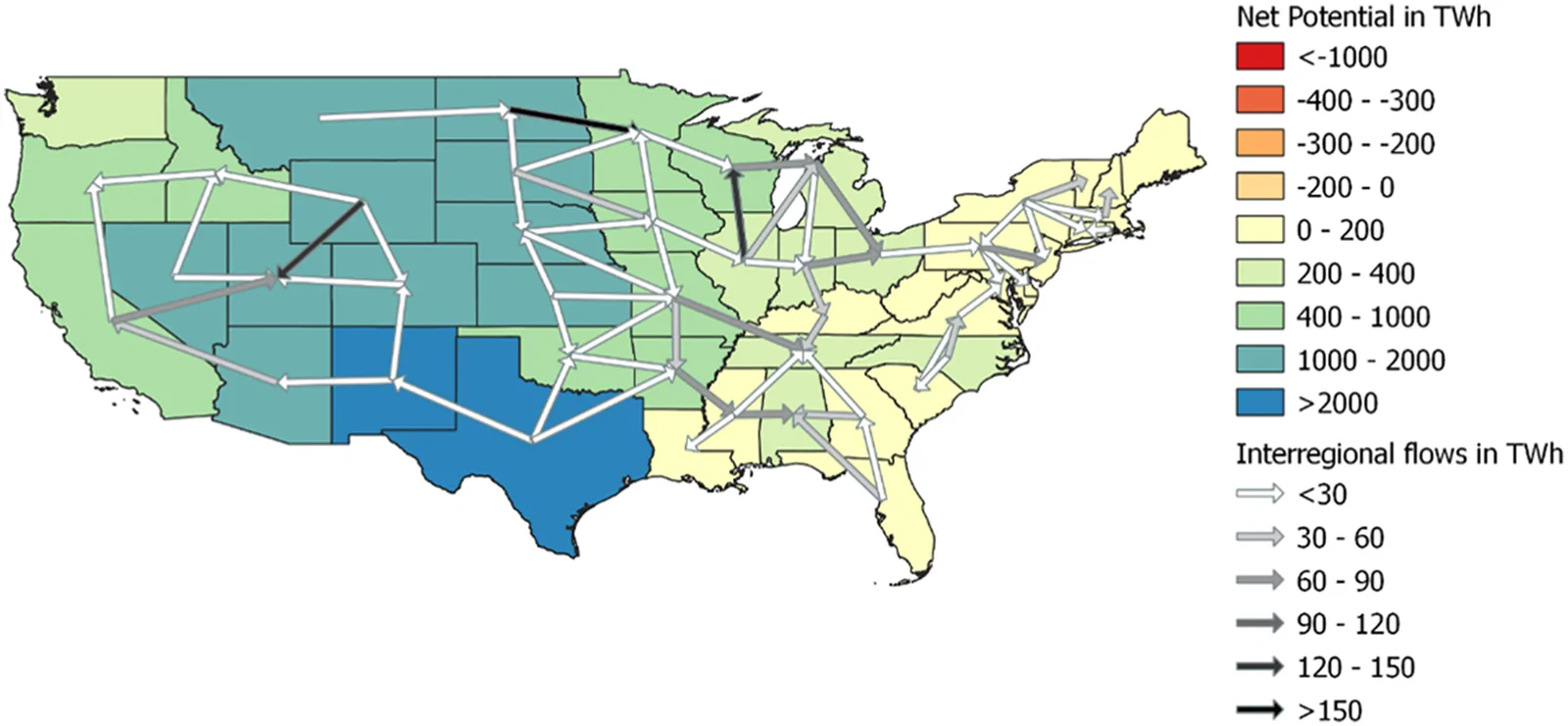
Net potential in the United States per state (TWh) This accounts for the generation and demand of electricity and hydrogen (converted into electricity equivalents), as well as cross-regional imports and exports. The color scale shows regional net potential, while the arrows -shown in grayscale-represent the magnitude of the electricity flows between states.

Figure 9 presents the renewable energy generation potential by cost step in comparison to the electricity demand for the United States. Texas stands out with more than 2,500 TWh of potential available at a generation cost of less than 20 €/MWh. Several Midwestern and Western states, including Kansas, Arizona, Wyoming, Colorado, and Nevada, also have substantial potential exceeding 1,000 TWh at the same cost threshold.

**Figure 9:**
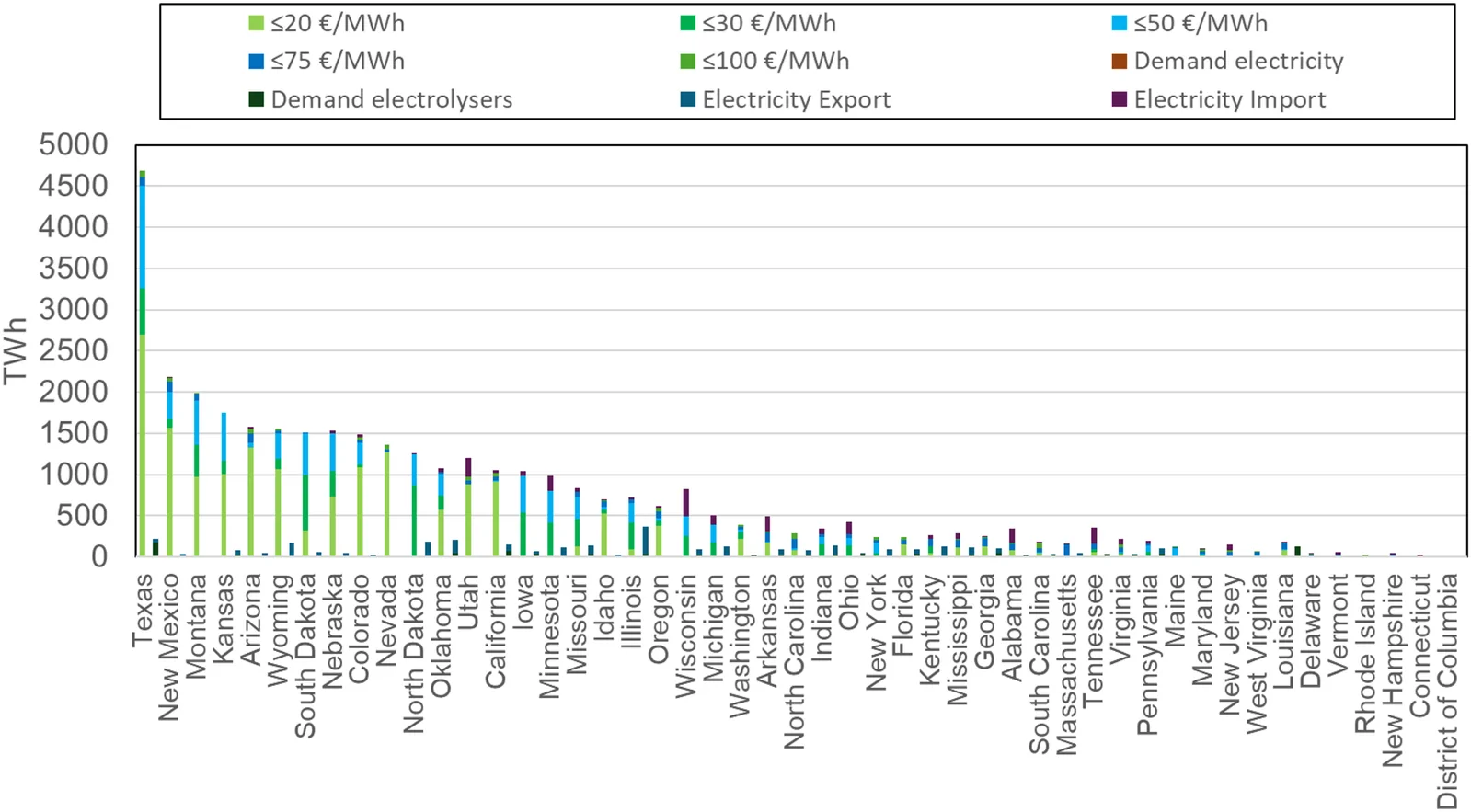
The potential for renewable electricity generation and the projected demand for electricity in the United States in 2050, broken down by state Generation potential is shown by the levelized cost of electricity (LCOE) ranges. Demand values include hydrogen demand converted into electricity equivalents. Inter-regional imports and exports are included in the regional net balance.

On the other end of the spectrum, states such as Connecticut, New Hampshire, Rhode Island, and Vermont exhibit the lowest potential for renewable energy. Nevertheless, these lower values are generally sufficient to meet local demand.

The inclusion of interstate electricity transmission allows for the redistribution of electricity from high-surplus states to those with lower surplus levels, thereby enhancing overall system balance and regional adequacy.

### Global hydrogen import potential

The scale of hydrogen imports is calculated based on the demand-supply gap. Table 2 presents the resulting median, mean, high-import, and low-import cases from the meta-analysis. The literature-based hydrogen import requirement ranges from 128 to 1,255 TWh across the low- and high-import cases, with a median estimate of 458 TWh and a mean estimate of 438 TWh. The results show that the European countries are mostly driving the need for global hydrogen imports, whereas the contribution of the Asian region remains small in the median and mean cases but increases under the high-import case. The estimated import range is sensitive to the filtering criterion, varying from 112–1,588 TWh without filtering to 128–1,255 TWh with the ±1.5 SD filter and 201–990 TWh with the ±1 SD filter (Table S6).

**Table 2:** Literature-based hydrogen import requirement in 2050 (TWh), based on the mean ± 1.5 SD filtered dataset

Country/Region	Median	Mean	High import case	Low import case
European region^c^	454^a^	420	866	128
Germany	33	71	198	−121^b^
Italy	131	82	279	−53
Netherlands	95	80	139	1
Belgium	125	120	152	94
Austria	27	26	35	14
Czechia	43	40	64	18
Asian region	4	18	389	0
Japan	−83	−104	216	−415
South Korea	−28	−24	25	−71
Thailand	4	18	64	−17
Indonesia	−30	−238	69	−426
Vietnam	−55	−38	15	−136
Total import projection^c^	458	438	1,255	128

See Tables S4 and S6 for sensitivity analyses using alternative filtering approaches.

a Positive values refer to the import demand of each country.

b Negative values refer to the additional hydrogen production potential after covering domestic demand.

c For regional and total import requirements, negative country-level values were set to zero because they represent domestic surplus rather than negative imports.

The Chinese provinces identified as deficit regions have a combined hydrogen demand of around 550 TWh. Depending on the share met through international supply, this could correspond to 0, 275, or 550 TWh of additional hydrogen imports for import shares of 0%, 50%, and 100%, respectively. By contrast, all U.S. states possess sufficient renewable potential to meet their own demand. Consequently, hydrogen imports are expected to be negligible or non-existent.

The long-term outlook for hydrogen could shift with the implementation of targeted policy measures, which essentially concern international commitment to climate protection goals. However, it remains unlikely that the hydrogen economy will scale up to a global level, enabling international trade, as hydrogen transport costs will be significantly higher than those of LNG transportation. In contrast to the oil market, the natural gas market is therefore strongly characterized by regional structures, and this will be even more pronounced in a hydrogen market due to the significantly higher transport costs of hydrogen.

## Discussion

The meta-analysis indicates that international hydrogen imports will increase but remain limited before 2050. In line with our findings, analyses by the Hydrogen Council, McKinsey,^8^ IEA,^9^ and IRENA^32^ suggest that large-scale multinational projects are unlikely before 2030, constrained by weak investment frameworks and uncertainty in energy demand and decarbonization pathways.

While there is a clear focus on global hydrogen export projects, a significant shortage of committed off-taker countries remains.^17^ A similar trend applies to the numerous potential hydrogen-exporting countries, given the vast renewable energy potential available across many regions. This contrasts sharply with conventional energy markets, such as oil and natural gas, where hydrocarbon deposits are geographically concentrated in a few regions. As a result, traditional energy markets exhibited higher market values and intensified competition among consumers. In the emerging hydrogen paradigm, the dynamic may shift toward the consumer side, compelling suppliers to reduce costs and offer additional environmental benefits.

At the national level, country-specific hydrogen strategies indicate a growing demand for renewable-based electrolytic hydrogen by 2030. However, despite the large renewable energy potential, the required global electrolyzer capacity remains insufficient, as scaling up production necessitates expanded manufacturing capabilities and requires up to a decade for feasibility studies and project preparation.^78^ Hydrogen production pathways vary, and some countries, under their decarbonization policies, accept hydrogen produced via steam methane reforming with carbon capture and storage as well as hydrogen from nuclear-based electricity. This could significantly reduce the scale of hydrogen traded on a global level, as countries will prioritize local solutions.

The discussion is also divided into three parts: a meta-analysis of European and Asian import-oriented countries, case studies on China and the U.S., and an examination of global hydrogen import potential.

### Meta analysis

Many global studies assessing the prospects of the hydrogen market tend to overlook the region-specific hydrogen production potential in import-oriented countries. This paper reviews literature to identify trends shaping major players as hydrogen importers, exporters, or both. The findings are considered in the context of regulatory and policy frameworks, in which import targets for some countries appear to be overestimated, sometimes by severalfold, compared with system studies aiming for the lowest energy system costs for the same countries. This mismatch between policy-driven targets and energy system studies hinders efficient decision-making. While ambitious targets risk unnecessary investment, actual developments in energy transition may also fall short of even the most conservative forecasts.

#### European countries

The meta-analysis of selected import-oriented countries in the European Union (Germany, Italy, the Netherlands, Belgium, Austria, and the Czech Republic) revealed a consistent trend: hydrogen demand exceeds the local renewable hydrogen production potential, indicating that they can potentially be strongly import-oriented (Figure 3). The results are shaped by differences in the availability of literature. For example, Germany has the largest number of collected studies (Table S3), spanning economic hydrogen production potential, policy and national strategies, and optimization analyses. For the Czech Republic and Austria, most studies are energy system analyses, which may underestimate the total hydrogen production potential but indicate the economically competitive volumes that can be produced domestically.

Several studies suggest that Europe could achieve energy independence by fully utilizing its high renewable potential within a pan-European energy system,^13^^,^^38^^,^^79^^,^^80^ which is consistent with our findings. The import-oriented countries are located mainly in northwestern and central Europe, characterized by high energy demand, particularly in the industrial sector.^81^ Other European countries, such as Spain, could supply renewable hydrogen to the deficit countries, complementing results from Lenivova,^80^ where the Spanish economic renewable hydrogen potential can be comparable to that of Morocco. Hydrogen imports could also originate from the Middle East and North Africa^33^^,^^38^^,^^82^ as well as other hydrogen production pathways that could influence the scale of imports required. For example, Norway and the UK are basing their hydrogen strategies on the production of blue hydrogen (produced through steam methane reforming (SMR) with carbon capture and storage (CCS)-based technology) and renewable hydrogen for export to countries such as Germany and the Netherlands. In the near term, blue hydrogen is considered more promising than electrolytic hydrogen, due to its lower costs and earlier deliveries.^83^

#### Asian countries

The meta-analysis for the selected Asian countries revealed different results from those for the European countries, where the potential for hydrogen import is less evident (Figure 4). While several studies position Japan and South Korea as H_2_ importers, our findings suggest that there is sufficient technical renewable energy to meet demand and decarbonize their energy systems. Nevertheless, national strategies in South Korea, Japan, Vietnam, and Singapore project substantial import volumes, highlighting a divergence between technical potential and policy ambitions.

Consistent with these projections, several studies examine hydrogen supply chains from Australia to Japan or to South Korea.^12^^,^^13^^,^^84^^,^^85^^,^^86^ The PtX Atlas developed by Pfenning et al.,^12^ estimates economic supply at €120–140/MWh, depending on the shipping carrier. Kim et al.^7^ report an LCOH of €192/MWh-H_2_ for the Australia-Korea route, with production and liquefaction accounting for €101 and 36/MWh-H_2_, respectively. Brändle et al.^13^ find that due to geographical constraints and limited infrastructure, imports would likely be shipped, adding €59–87/MWh-H_2_ in transport costs, making domestic hydrogen in Japan (optimistic €51/MWh-H_2_ by 2050) potentially competitive. The HYPAT project^11^ estimates the economic potential for hydrogen production in Japan to be less than 100 TWh under 90€/MWh (or 3 €/kg), supporting the possibility of competitive domestic supply. However, avoiding imports entirely would double Japan’s power demand by 2050 compared with 2019. This aligns with Burandt (2021),^87^ who emphasizes that even in countries with poor renewable resources, domestic electrolytic hydrogen can play a balancing role in the energy system. Overall, our results indicate that while imports may support diversification and security of supply, they are unlikely to be the primary driver for achieving net zero in these Asian economies.

South Korea is often identified as a potential net importer of hydrogen, with a national strategy projecting demand in the mobility, power generation, and industrial sectors. Renewable hydrogen production potential (mainly PV, onshore, and offshore wind) appears sufficient to meet demand by 2050; however, costs are expected to remain high due to poor wind conditions and limited land availability.^88^ In recent years, South Korea has pursued international hydrogen cooperation, signing memorandums of understanding (MoUs) with Canada, the U.S., and Australia.^89^ The country also aims to secure a niche in fuel cell and fuel cell electric vehicles (FCEVs) manufacturing and become a global supplier.

In Indonesia, high geothermal and hydro resources can provide stable baseload renewable electricity, complemented by extensive PV potential of ∼1,200 TWh.^90^ By contrast, wind potential is the lowest among the Philippines, Thailand, and Vietnam. Despite this substantial renewable base, large-scale hydrogen production would require additional storage, including batteries and underground hydrogen storage.^90^ Similarly, Thailand, the Philippines, and Vietnam each have significant PV potential of 300–500 TWh, which is sufficient to meet domestic hydrogen demand, while their wind resources remain limited and costly due to low full-load hours (<600 FLH). These patterns highlight how resource profiles, rather than total renewable potential alone, influence the feasibility of hydrogen production across Southeast Asia.^90^

### Focus countries

This subsection discusses our results in the context of China and the United States, highlighting their respective needs for hydrogen imports.

#### China case study

In recent years, China has been one of the countries with the most significant expansion of renewable energy technologies. However, the uneven geographical distribution of RE resources across its territory has created substantial regional imbalances between generation and demand, particularly between the resource-rich northwestern regions and the high-demand eastern and coastal provinces. This discrepancy necessitates the development of large-scale transmission infrastructure to facilitate the transfer of electricity from production centers to consumption centers.

In addition to the RE technologies considered in this study, the Chinese government has announced plans to install up to 500 GW of nuclear power capacity. Assuming an FLH value of 7,500 h per year, this capacity would generate an annual electricity output of around 3,750 TWh. According to our analysis, the cumulative electricity deficit in the eastern regions is estimated to be around 7,000 TWh, indicating that the planned nuclear capacity alone could potentially supply roughly half of this deficit. Additionally, although the national policy does not set an explicit target for CCS deployment, it is mentioned as one of the key development fields and may potentially play a significant role in China’s decarbonization and possibly also in hydrogen supply.

Furthermore, the hydrogen demand in the deficit regions is estimated to be around 550 TWh. This demand may need to be partially met through hydrogen imports, potentially from regions with abundant resources, such as Australia, where sufficient renewable electricity generation exists to support large-scale hydrogen production and export.

The calculated offshore wind potential in our study amounts to slightly above 900 TWh across all provinces, whereas other studies report values from 2.5–4.2 PWh to around 12 PWh.^91^^,^^92^ A key difference is that our assessment considers an economic potential capped at 100 €/MWh and limits offshore areas to a water depth of up to 50 m, while Song et al.^91^ consider depths of up to 60 m. However, as noted by Song et al.,^91^ areas at a depth of 60 m are located further from the coast, making them less suitable for local electricity and hydrogen supply. A recent farm-level assessment estimates China’s offshore wind potential at 2.5–4.2 PWh/year, accounting for realistic turbine layouts and wake losses. It also highlights the higher costs and greater potential densities of deep-water floating wind compared with nearshore bottom-fixed wind.^92^ As offshore floating turbines continue to develop in the future, water depth is expected to become a less limiting factor.^66^ This progress could increase the available resource potential and further reduce China’s hydrogen import requirement.

Our results are consistent with those of Meng et al.,^93^ who clearly identify regions with high renewable energy potential. This spatial distinction is delineated by the so-called Hu’s hydrogen line. As illustrated in Figure 6, our results similarly highlight the differentiation between regions rich and poor in renewable energy resources. Meng et al.^93^ further suggest that areas with abundant renewable potential could serve as exporters, supplying regions such as Europe.

#### U.S. case study

In contrast to China, the United States does not exhibit clearly defined regions with structural energy deficits. However, states along the East Coast utilize nearly the entirety of their available renewable potential up to a cost threshold of 100 €/MWh. Given this limited in-state potential, these regions may increasingly rely on electricity imports from areas with more abundant resources. One such option is strengthening interconnections with Midwestern states, which offer significantly lower-cost renewable energy potential.

Another strategic alternative, though not considered in this paper, is the further expansion and utilization of existing transmission interconnections with Canada. Today, the total capacity of these cross-border connections stands at approximately 18 GW. The electricity exchange, both exports and imports, totals 77 TWh. The grid is expected to be expanded by five additional GW. The eastern coast of the United States, which is one of the regions with lower available RE potential, is currently connected to the province of Quebec. This permits the export of renewable electricity from hydroelectric plants to the United States. However, no plans currently exist to expand the interconnection capacity with the United States and Quebec. Quebec plans to expand its hydro generation capacity by 9 GW in 2035 and strengthen its internal interconnection capacity. This may indirectly benefit the East Coast as a larger amount of clean electricity may be available to be transported.^94^^,^^95^ Similarly, Mexico is closely connected to the United States. Currently accounting for only 0.14% of electricity demand, this connection could be strengthened in the future.^96^

In addition to the current and planned electricity interconnections, the region has also begun planning a common framework for hydrogen trade. In 2023, Mexico, the United States, and Canada pledged to explore standards for clean hydrogen. Such standards will facilitate the exchange of clean hydrogen within the region, enabling the creation of a regional hydrogen market. This may be particularly relevant for Canada, as it is closer to regions that may need to rely on high-cost renewable energy sources.

In terms of non-variable baseload capacity, the United States had approximately 94 GW of installed nuclear power as of 2022, with plans underway to substantially expand this figure, potentially tripling or even quadrupling it through the deployment of new plants across the country.^97^ A recent U.S. Department of Energy assessment highlights the siting potential of both retired coal plants and existing or retired nuclear facilities, assuming the use of 600 MW nuclear units: retired coal sites could accommodate an estimated 174 GW of new capacity, while nuclear sites could host an additional 60 GW.^98^ These locations offer the advantage of pre-established community acceptance of power generation infrastructure, thereby reducing potential siting and permitting barriers. Several states, including Pennsylvania, New York, Virginia, and Georgia, were identified as having considerable nuclear potential; while their renewable resources are sufficient to meet future needs, their margins are narrow, making nuclear a valuable complement. Other states, such as Texas and Ohio, could also benefit, with Texas positioned to become a major energy supplier by leveraging both its nuclear siting potential and its vast renewable resources.

### Global hydrogen import potential

Based on the results of the meta-analysis, the global hydrogen import requirement is estimated to range between 128 and 1,255 TWh. This accounts for a maximum of 10% of the projected global demand for hydrogen. This estimate is substantially lower than the trade volumes reported in the literature. However, the estimates are based on different assumptions. Our results represent the global hydrogen import requirement under domestic-resource prioritization, whereas other studies include economic trade between countries with sufficient domestic resources. For comparison, McKinsey & Company estimates that by 2050, demand for clean hydrogen can reach 12.5 PWh, of which more than half (6.3 PWh) will be transported over long distances. In 2050, less than half of the traded hydrogen will be delivered via long-distance pipelines, mainly concentrated in the North American region, North Africa, Europe, and China, while the rest will be realized in the form of shipments of hydrogen derivatives.^8^ Fakhreddine et al.^99^ estimates that global hydrogen demand will reach 234 Mt (7.8 PWh) by 2050, with around 30% (2.34 PWh) expected to be traded internationally, substantially exceeding our estimated import requirement of 128–1,255 TWh.

Despite the potential for renewable and nuclear expansion, our analysis shows that China’s eastern regions will continue to experience an energy deficit by 2050. Hydrogen imports could partly alleviate these regional imbalances. Depending on the share of deficit-region hydrogen demand met internationally, imports could range from 0 to 550 TWh, with 275 TWh corresponding to an illustrative 50% import share. The situation in the United States is markedly different: by 2050, no states are projected to face an energy deficit when accounting for renewable resources and planned transmission line expansions. In addition, the country has ambitious nuclear expansion plans and a strategic interconnection with Canada, which together imply that its demand for hydrogen imports will remain minimal.

It is essential to note, however, that these findings rely on the assumption of a fully decarbonized global energy system by 2050, which requires aggressive renewable deployment worldwide. The analysis assumes that countries not identified as importers lack the incentive to export hydrogen. However, in practice, this assumption may not be applicable, as energy security concerns or comparatively lower production costs in neighboring regions could motivate export activity.

The analysis indicates that European countries such as Germany, the Netherlands, Belgium and Italy will remain import-oriented, whereas some Asian countries, including South Korea, Indonesia and Vietnam possess sufficient renewable energy potential to cover their decarbonization needs. The case studies of China and the U.S. further highlight the importance of regional differences between renewable energy availability and demand, as well as the role of transmission infrastructure in balancing these differences. Similar regional dynamics could unfold in other large economies, including India, Russia, and Mexico, which can be investigated in future research.

Overall, the hydrogen market is likely to remain regional and interregional rather than global due to the widespread distribution of renewable resources with comparably low electricity production costs, as well as the high transportation costs of hydrogen, both compared to conventional fossil fuel markets. In contrast, synthetic fuel markets may develop toward more extensive global trade, with hydrogen production and subsequent conversion steps concentrated in regions with the lowest-cost renewable energy, and hydrogen-based intermediate or final products traded internationally. This could be facilitated by the transport properties of synthetic fuels, their similarities to conventional fossil fuels, and the possibility of using established fuel infrastructure.

### Limitations of the study

This study has limitations regarding the assessment of country-specific hydrogen demand, domestic supply potential, and future import requirements.

The country screening is based on the net energy balance between renewable electricity generation potential and projected domestic primary energy demand for 2050 under a net-zero transition scenario. These quantities are not directly comparable, as primary energy demand includes conversion losses as well as non-electric energy carriers. Therefore, this comparison should be interpreted as a first-order indicator of domestic renewable energy resource sufficiency rather than as a detailed national energy balance. In a highly electrified climate-neutral energy system, the majority of hydrogen production is expected to originate from renewable electricity via electrolysis, making renewable electricity potential a suitable indicator for the initial screening.

The analysis focuses on hydrogen import demand and excludes cases involving synthetic fuel trade. Additionally, as the analysis is focused on the import demand, it does not cover hydrogen transport methods; the work by Pieton et al.^100^ provides insights on long-distance transport. Hydrogen demand is considered based on final hydrogen consumption. Consequently, the synthetic fuel market is not considered in this study, which could present a fundamentally different global landscape compared to hydrogen. Further information on synthetic fuel trading and export- and import-oriented countries for PtX/L products is addressed in the article by Galimova et al.^19^ Furthermore, considerations regarding security of supply were not the focus of this study.

The results may be influenced by the number and type of studies available for each country. Overall, there is a clear preponderance of studies for countries such as Germany, Italy, Japan, and South Korea, reflecting substantial research funding programs in recent years. This highlights the need for more country-specific analyses in regions where few studies currently exist. Further research is required to represent the global hydrogen market in more detail and capture countries’ cross-border trade interdependencies in global energy system models. For the in-depth case study analysis, a clear limitation is the lack of data on hydrogen pipeline plans in the United States. However, the country is well-positioned, and intra-state hydrogen exchanges are unlikely to significantly affect the national balance.

Our study focuses on the economic potential for renewable hydrogen production in importing countries, excluding any capacity for other low-carbon hydrogen production pathways. When these are considered, they will further reduce renewable hydrogen import volumes. Other constraints, such as the potential for geological hydrogen storage and existing gas infrastructure, are also not considered and may influence the allocation of renewable hydrogen production in countries that are both exporters and importers. Additionally, advances in hydrogen certification could influence the development and implementation of potential hydrogen trade agreements, which were beyond the scope of this study.

## Resource availability

### Lead contact

Requests for further information and resources should be directed to and will be fulfilled by the lead contact, Veronika Brooks (veronika.brooks@ieg.fraunhofer.de).

### Materials availability

This study did not generate new unique materials.

### Data and code availability

• The literature database (Literature_database.xlsx), containing 388 collected literature values for hydrogen demand and domestic hydrogen supply potential, study classifications, references, and processed meta-analysis results, has been deposited and is publicly available as of the date of publication at Mendeley Data: https://doi.org/10.17632/3vvdkgryr4.1.
• The preprocessing and reproducibility script (reproduce_meta_analysis.py), used for filtering the literature data and calculating the hydrogen import requirement estimates, has been deposited and is publicly available as of the date of publication at Zenodo: https://doi.org/10.5281/zenodo.22171298.
• Any additional information required to reanalyze the data reported in this paper is available from the lead contact upon request.

## Acknowledgments

This project was funded by the German Ministry of Education and Research (10.13039/501100002347BMBF) within the research program „HYPAT: Globaler H2-Potenzialatlas – nachhaltige Standorte in der Welt für die grüne Wasserstoffwirtschaft von morgen“ (funding reference number 03SF0620A). The authors are solely responsible for the content of this publication.

## Author contributions

Conceptualization, V.B., J.F.G., C.N., and M.W.; data curation, V.B. and J.F.G.; formal analysis, V.B., J.F.G., L.Z., V.P.M., C.N., D.M., and M.W.; investigation, V.B. and J.F.G.; methodology, V.B., J.F.G., L.Z., V.P.M., D.M., and M.W.; software, V.B. and J.F.G.; validation, V.B., J.F.G., L.Z., and V.P.M.; visualization, V.B. and J.F.G.; writing – original draft, V.B. and J.F.G.; writing – review and editing, V.B., J.F.G., L.Z., V.P.M., C.N., D.M., and M.W.; funding acquisition, M.W.; project administration, M.W.; resources, M.W.; supervision, D.M. and M.W.

## Declaration of interests

The authors declare no competing interests.

## Declaration of generative AI and AI-assisted technologies in the manuscript preparation process

During the preparation of this work, the authors used DeepL and ChatGPT to check the wording and improve the readability of the manuscript. After using this tool/service, the authors reviewed and edited the content as needed and take full responsibility for the content of the publication.

## STAR★Methods

### Key resources table

**Table:** 

REAGENT or RESOURCE	SOURCE	IDENTIFIER
**Deposited data**		

Literature database and processed meta-analysis data	This paper	Mendeley Data: https://doi.org/10.17632/3vvdkgryr4.1
Global renewable energy potential data	Kleinschmitt et al.^101^	HYPAT renewable energy potential maps
National Transmission Planning Study	NREL	National Transmission Planning Study
Hydrogen production cost/ potential data	Mendler et al.^29^	https://doi.org/10.1016/j.ijhydene.2024.07.320

**Software and algorithms**		

Meta-analysis preprocessing and reproducibility script	This paper	Zenodo: https://doi.org/10.5281/zenodo.22171298
Renewable Potential Calculator 2.0/ Enertile	Franke et al.^102^	https://doi.org/10.1016/j.renene.2024.120376

### Experimental model and study participant details

This study does not involve experimental models, human participants, animals, plants, or microbial strains.

### Method details

This section outlines the methodological steps used in this paper. First, we describe the screening of countries with potentially limited domestic renewable resource availability relative to projected energy demand in 2050. Second, we present the methodology used to conduct the meta-analysis. Finally, we elaborate on the methodology applied for the case study calculations of China and the United States. The overall methodology is shown in Figure 1.

#### Country screening

The first step in our study is to identify countries with future hydrogen import needs, as shown in Figure 1. We focus on countries that may face an energy deficit when comparing projected primary energy demand with their renewable electricity generation potential in 2050. Our analysis prioritises electricity because most processes are expected to be electrified to achieve decarbonisation.^103^ Electricity is crucial for producing electrolytic hydrogen, the primary focus of this paper. Therefore, in this section, we first describe the methodology used to determine the renewable electricity generation potential, followed by the calculation of energy demand projections. Finally, we outline the process for selecting the import countries (see step 2 on the left side of Figure 1).

#### Renewable electricity generation potential

Renewable electricity generation potential is assessed for utility-scale PV, concentrated solar power, wind onshore, and wind offshore technologies using the Renewable Potential Calculator 2.0 of the Enertile model on a global scale.^102^ The model divides the Earth’s surface into approximately 12 million tiles, each 6.5 x 6.5 km^2^. Weather data are assigned to each tile to calculate hourly generation time series for each technology, using ECMWF ERA5 reanalysis data for 2010.^104^ To determine the potential installable capacity, land types are assigned to each tile based on the GlobCover 2009 dataset.^105^ Rooftop PV was not included in the calculation, as the electricity produced by this technology is difficult to allocate for large scale hydrogen production. Subsequently, land-use factors are then applied to each land type, indicating the proportion of land that can be used for each technology. The model excludes protected areas classified as Ia, Ib, and II by the International Union for Conservation of Nature and Natural Resources.^106^ Results from individual tiles are aggregated as a weighted average and systematically categorised into steps according to their levelized cost of electricity (LCOE), with step 0 representing the lowest cost. 2018 is used as base year for LCOE calculation. Further details of these calculations can be found in the literature.^29^^,^^101^^,^^102^

#### Global energy demand estimation

The future domestic energy demand was approximated using the projected primary energy demand from the Global Energy and Climate Outlook 2020 (GECO) scenario 1.5 °C,^46^ consistent with the goals of the Paris Agreement, following the methodology proposed by Breitschopf et al.^107^ Building on the JRC Global Energy and Climate Outlook 2020,^46^ this study provides projections of future primary energy consumption for 172 countries worldwide. For countries not covered by the JRC outlook, country-level estimates were derived using a disaggregation methodology to ensure consistent global coverage.^107^ This demand metric was used solely for the purpose of the initial global screening and does not represent future electricity demand.

#### Identification of candidate countries

To identify countries with potentially limited domestic renewable resource availability, we subtracted renewable electricity generation potential from projected domestic energy demand, following the methodology proposed by Breitschopf et al.^107^ Using primary energy demand in the screening may result in larger resource gaps than a final-energy comparison and therefore retain more countries as potential importers. Locations with unrealistically high generation costs were excluded by applying a cost threshold of 100 €/MWh to the renewable electricity generation potential, according to the LCOE values calculated with the Enertile model.^101^ This threshold allows for the inclusion of offshore wind potential, which, despite higher capital costs, offers advantages such as fewer acceptance issues and additional generation potential. To assess the sensitivity of this assumption, the same analysis was conducted using electricity cost thresholds of 50, 75 and 100€/MWh, with the corresponding candidate countries and gaps between renewable electricity potential and projected energy demand reported in Figure S1 and Table S1 of the Supplemental Information. Lower thresholds reduce the renewable resource base and therefore increase the magnitude of the screening gaps. Across the 16 countries retained for the subsequent literature analysis, the aggregate resource gap decreases from 14,310 TWh at 50 €/MWh to 12,118 TWh at 75 €/MWh and 11,324 TWh at 100 €/MWh. However, all 16 countries exhibit a resource gap under each threshold; the sensitivity therefore affects the magnitude of the gap rather than their selection. The analysis assumes that countries prioritise domestic renewable resources and that the global energy system follows a 1.5°C–2.0 °C decarbonisation pathway by 2050, in line with the Paris Agreement. Countries with limited net renewable potential were identified as candidates for further investigation and listed in Table 1. By excluding countries with resource gaps smaller than 30 TWh, we identified the regions with the most significant energy deficits: Central Europe, Eastern Asia and Southeast Asia. These areas will be referred to as the European and Asian regions thereafter. Consequently, the analysis focuses on these two regions. Trinidad and Tobago and Bahrain were excluded because they fall outside these categories. The goal of this screening was to establish a list of countries which have a limited renewable energy resource and therefore are likely candidates to become net H_2_ importers for subsequent literature-based assessment.

#### Meta-analysis

The potential hydrogen import requirement of the selected 16 countries was evaluated through a meta-analysis based on two categories: hydrogen demand projections and renewable hydrogen supply potential in 2050. Because the same 16 countries are retained under all three screening thresholds and the meta-analysis uses independently collected literature estimates, the screening threshold does not affect the literature-based aggregate import estimate. Countries were retained in the meta-analysis only if at least five estimates in each category remained after filtering. As shown on the left side of Figure 1, Step 2, the meta-study combines various study types (Tables S2 and S3), including studies on renewable hydrogen production potential, energy system analyses, and national strategies and roadmaps. We also added our own calculations for green hydrogen production potential based on the HYPAT project.^11^^,^^29^ The literature was collected according to the following criteria:

*Demand studies:* focus on year 2050; the collected studies considered the final hydrogen demand. The discrepancies in the assumptions for the hydrogen sectoral demand led to a wide spread in the future projections, see Table S2.

*Supply studies:* focus on year 2050; renewable hydrogen production potential, energy system studies with 100% renewable electricity, and economic hydrogen production potential. In the energy system optimisation studies, domestic hydrogen production represents the amount selected endogenously by the model as cost-competitive under the respective demand and scenario assumptions, see Table S2.

In total, 388 values were collected and are available in Data and Code availability section. All studies from the meta-analysis were categorised into five types to provide a better overview of the methodological differences between the collected values: economic potential, referring to renewable hydrogen production potential below a defined cost threshold of approximately 2–3€/kg (70–100€/MWh); energy system studies, referring to endogenous domestic renewable hydrogen production resulting from energy system optimisation; strategy, referring to national strategies, targets, and roadmaps related to renewable hydrogen production; demand, referring to national hydrogen demand projections; and other, referring to studies for which the methodology used to derive hydrogen production potential was not sufficiently described. The number and type of collected values for each country are reported in Tables S2 and S3. For the third step, we applied a mean ± 1.5 standard deviation (SD) filter to reduce the influence of extreme literature estimates on the hydrogen demand and supply distributions for each country. Comparisons between the unfiltered results and results using the ±1 SD and ±1.5 SD filters are provided in the Supplemental Information, Figures S2 and S3, as well as Table S6. Applying the ±1.5 SD filter reduced the influence of extreme literature estimates. Compared with the unfiltered results, country classifications based on median demand-supply balances remained unchanged, except for Thailand, which shifted from a small surplus (1 TWh) to a small import requirement (4 TWh). In the final step, differences between the quartiles of hydrogen demand and production potential were calculated to define lower and upper literature-based import cases, forming the basis for estimating the total global hydrogen import requirement.

#### In-depth analysis with case studies

Following the methodology description of the meta-study, this section presents the methodology for the in-depth analysis of the two case studies, China and the United States. China and the United States do not exhibit a national resource gap under any of the three screening thresholds and are therefore treated separately as case studies to assess subnational mismatches between renewable resources and demand.

The electricity and hydrogen demand for 2050 was calculated using the meta-study described in the previous section. The median values from this study were then distributed among US states and Chinese provinces based on their current electricity consumption shares.^108^^,^^109^ It is therefore assumed that these regional shares will remain constant by 2050. As regional electricity demand patterns may evolve by 2050, the resulting spatial balances are interpreted as indicative. Assuming a 70% efficiency, hydrogen demand was converted into electricity equivalents, enabling both energy carriers to be evaluated consistently.

The renewable energy potentials were aggregated at the province and state levels, as illustrated in Step 2 on the right-hand side of Figure 1. The values correspond to the same potentials described in Section 2.1.1, re-aggregated to these administrative units. For offshore wind, a share of the exclusive economic zone was assigned to each coastal state or region. The potentials were capped at 100 €/MWh. The case-study results are reported separately from the literature-based aggregate and therefore do not affect the headline import estimate. As a final step (Step 4, right-hand side of Figure 1), we consider electricity (U.S.) and electricity and hydrogen (China) transmission between the states and regions. For the United States, we consider the transmission capacities simulated in the “National Transmission Planning Study” from NREL. The scenario AC_DemMd_90by2035EP__core was selected.^110^^,^^111^ This scenario is a conservative one that permits new interregional connections but no DC transmission lines. For the case of China, we considered both planned high-voltage DC lines and hydrogen pipelines from different sources.^112^^,^^113^^,^^114^^,^^115^^,^^116^

Although India is identified as an importer in our preliminary study, given its size and the potential, the possible spatial mismatch between supply and demand, it is a similar case to the United States and China. However, it was excluded from the present analysis due to resource constraints. Future work could expand the scope to include India alongside China and the United States.

#### Calculation of global hydrogen import requirement

For all candidate countries, the literature-based hydrogen import requirement was calculated by comparing the distributions of projected hydrogen demand and domestic renewable hydrogen supply potential. The difference between the median values of demand and domestic supply was used as central literature estimate, while Q3 demand minus Q1 supply and Q1 demand minus Q3 supply were used to define high- and low-import literature cases, respectively. The mean case was calculated as the difference between mean demand and mean supply. The country-level results were then summed across the selected European and Asian countries to derive the total global hydrogen import requirement. Negative country-level values were set to zero, as they represent domestic supply exceeding demand rather than negative imports. The China and U.S. case-study results were evaluated separately and are not included in the literature-based aggregate.

### Quantification and statistical analysis

The literature-based analysis included 388 collected values of hydrogen demand and domestic hydrogen supply potential. The number of values (n) refers to the number of individual literature estimates collected for each country and category; the exact n for demand and supply is reported in the corresponding figure legends and Table S6 in Supplemental Information. Demand and supply values were analyzed independently and were not systematically paired by study. Distributions were summarised using the median, first quartile (Q1), and third quartile (Q3).

To reduce the influence of extreme cross-study estimates, the main analysis applied a ±1.5 standard deviation filter separately to the demand and supply distributions of each country. Results were compared with the unfiltered data and with a ±1.0 standard deviation filter; these comparisons are reported in Figures S2 and S3 and Tables S4 and S6. Additionally, the 28 JRC-derived hydrogen demand estimates were increased by 10% in a sensitivity analysis described in Methods S1 to account for excluded non-energy feedstock uses; further details are provided in Table S7.

## Supplemental information

Document S1. Figures S1–S3 and Tables S1–S7 and Methods S1, and supplemental references
