# Supplementary material for: Who will import hydrogen in 2050? Global assessment with China and U.S. case studies
Source: iScience. 2026 Nov 9;29(11):118175. doi: 10.1016/j.isci.2026.118175 (PMC13770709; doi:10.1016/j.isci.2026.118175)
Supplement: Supplementary file 1 [file mmc1.pdf]

iScience, Volume *29*

Supplemental information

Who will import hydrogen in 2050? Global assessment with China and U.S. case studies

Veronika Brooks, Joshua Fragoso García, Lin Zheng, Viktor Paul Müller, Christoph Nolden, Dominik Möst, and Martin Wietschel

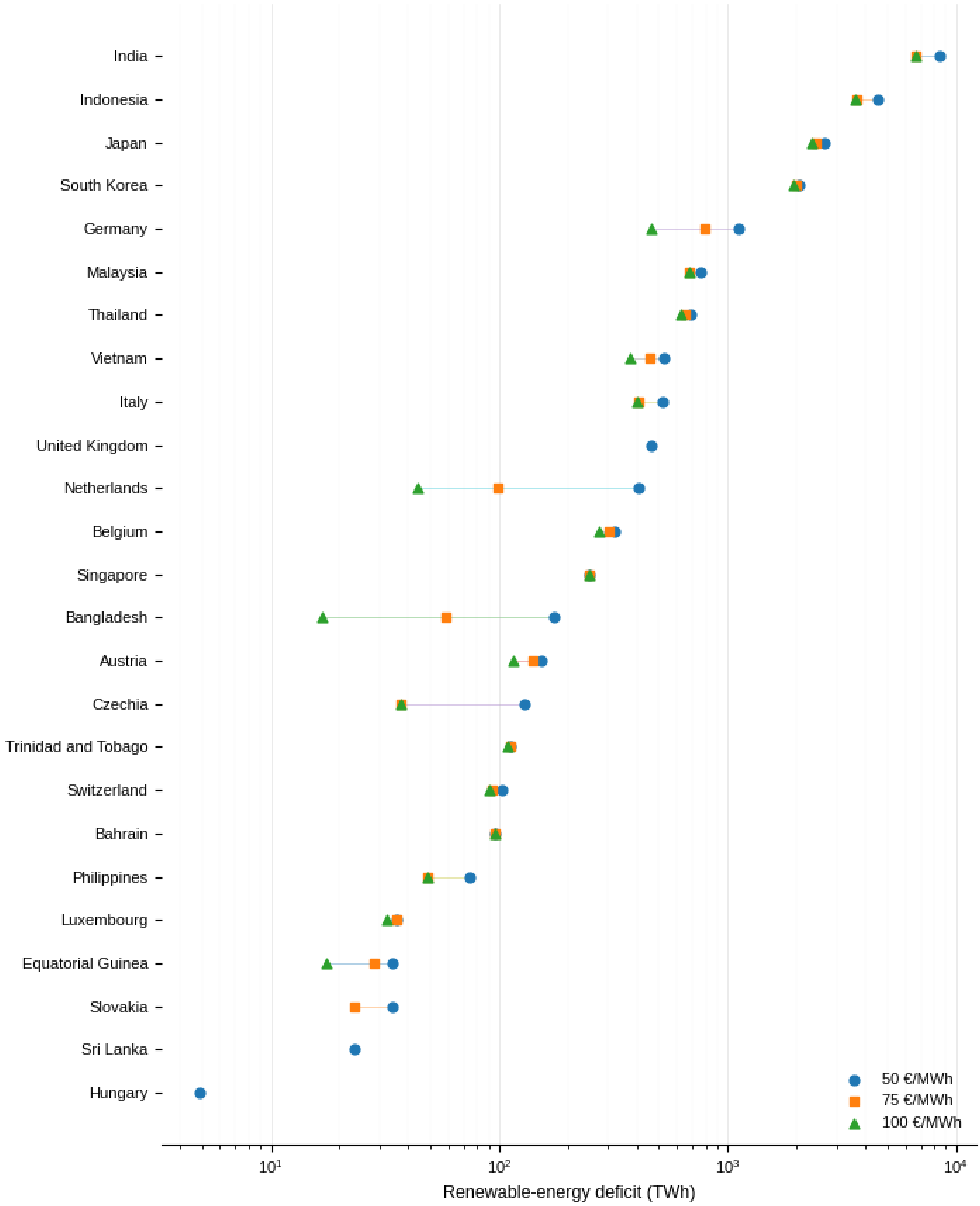

Figure S1. Sensitivity of the country screening to the renewable electricity cost threshold, related to Table 1.

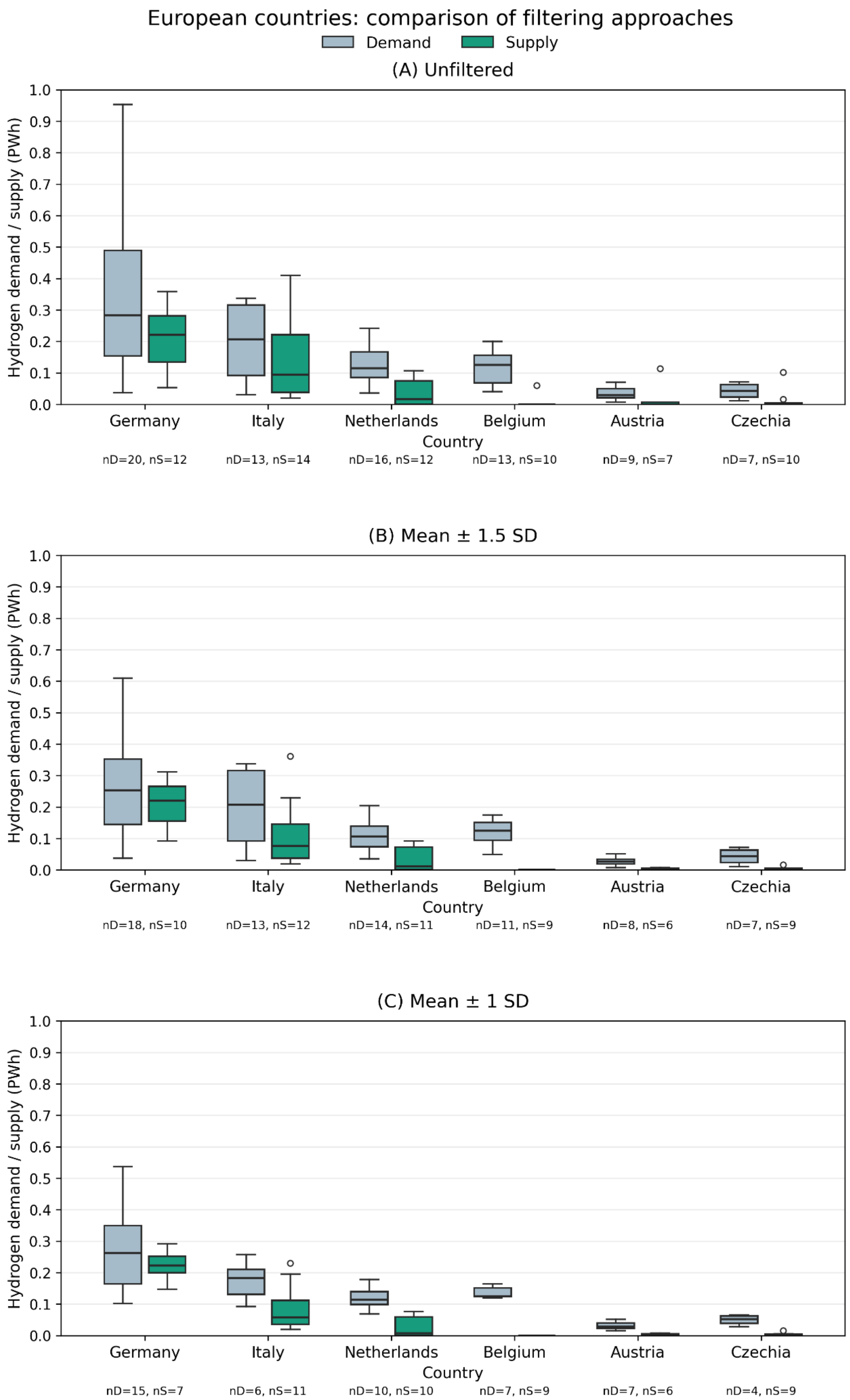

Figure S2. Literature estimates of hydrogen demand and domestic renewable hydrogen supply potential in European countries in 2050 under alternative filtering approaches, related to Figure 3. (A) Unfiltered literature estimates. (B) Estimates retained after applying the mean ±1.5 SD filter used in the main analysis. (C) Estimates retained after applying the mean ±1 SD filter.

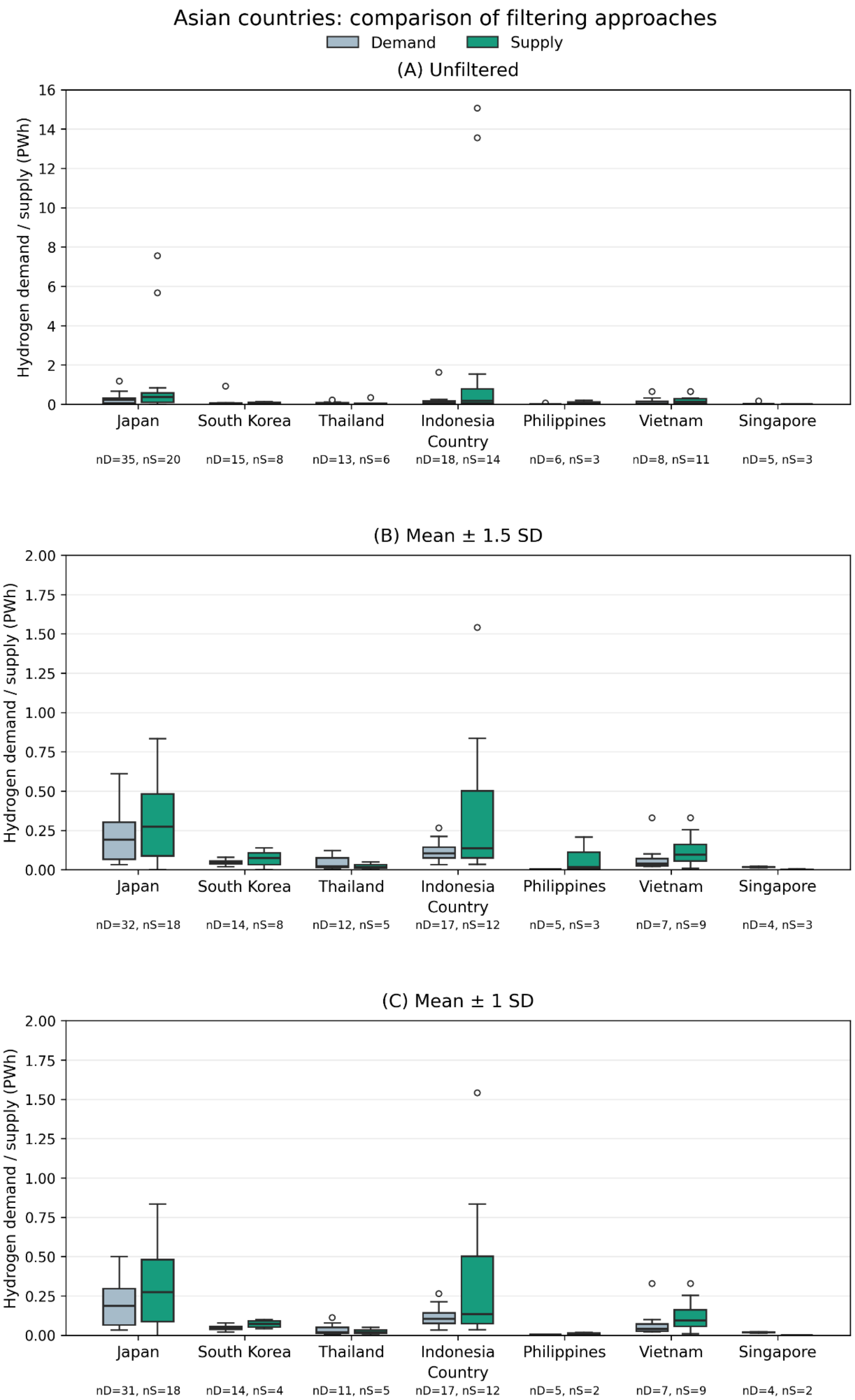

Figure S3. Literature estimates of hydrogen demand and domestic renewable hydrogen supply potential in Asian countries in 2050 under alternative filtering approaches, related to Figure 4. (A) Unfiltered literature estimates. (B) Estimates retained after applying the mean ±1.5 SD filter used in the main analysis. (C) Estimates retained after applying the mean ±1 SD filter.

Table S1. Country-level resource gaps (in TWh) under renewable electricity cost thresholds of 50, 75, and 100 €/MWh, related to Table 1.

Country	Resource gap under 50 €/MWh	Resource gap under 75 €/MWh	Resource gap under 100 €/MWh	Reduction from 50 to 100 €/MWh
India	8426	6666	6665	20.90%
Indonesia	4521	3669	3621	19.91%
Japan	2653	2430	2334	12.04%
South Korea	2063	1989	1939	6.02%
Germany	1111	792	464	58.26%
Malaysia	762	678	678	11.05%
Thailand	686	647	622	9.44%
Vietnam	529	454	376	28.85%
Italy	516	408	402	22.21%
United Kingdom	465	-	-	-
Netherlands	408	99	44	89.19%
Belgium	318	303	272	14.39%
Singapore	248	248	248	0.01%
Bangladesh	175	58	17	90.35%
Austria	153	140	115	24.70%
Czechia	129	83	37	71.43%
Trinidad and Tobago	113	111	109	2.99%
Switzerland	103	93	91	11.10%
Bahrain	95	95	95	0.00%
Philippines	74	49	49	34.41%
Luxembourg	36	36	32	9.46%
Equatorial Guinea	34	28	18	48.60%
Slovakia	34	23	-	-
Sri Lanka	23	-	-	-
Hungary	5	-	-	-

Table S2. Classification of study types included in the literature-based meta-analysis, related to Figures 3 and 4.

Type of study	Definition
Economic potential	The renewable hydrogen potential under 2-3 €/kg (70-100 €/MWh) threshold
Energy system study	Endogenous domestic renewable hydrogen supply as a result of the optimisation model
National strategy	National strategies, targets, and roadmaps related to renewable hydrogen production
Demand study	National hydrogen demand
Other	Other types of studies, where the methodology of obtaining the hydrogen production potential is not available

Table S3. Overview of the number of values collected per region, country and study type, related to Figures 3 and 4.

Region	Country						
		economic potential	system analysis	strategy	demand	other	Total
Asia	Indonesia	4	8	1	18	1	32
Asia	Japan	5	14	1	35	0	55
Asia	Philippines	1	1	0	6	1	9
Asia	Singapore	1	1	0	5	1	8
Asia	South Korea	5	1	2	15	0	23
Asia	Thailand	1	3	0	13	2	19
Asia	Vietnam	2	6	2	8	1	19
East Asia	China	7	0	0	18	3	28
Europe	Austria	1	6	0	9	0	16
Europe	Belgium	4	6	0	13	0	23
Europe	Czechia	4	6	0	7	0	17
Europe	Germany	4	8	0	20	0	32
Europe	Italy	6	8	0	13	0	27
Europe	Netherlands	1	11	0	16	0	28
North America	USA	7	23	0	21	1	52

Table S4. Sensitivity of the aggregated hydrogen import requirement to the filtering approach, related to Table 2.

Filter	Country	Median case	Mean case	High-import case	Low-import case
Unfiltered	Total only import	470.75	450.40	1,587.93	112.03
±1.5 SD	Total only import	458.14	438.22	1,254.84	128.32
±1 SD	Total only import	428.24	423.81	990.00	201.36

Table S5. Countries excluded by the minimum sample-size criterion under alternative filtering approaches, related to Figures 3 and 4.

Filter	Region	Country	n Demand	n Supply	Reason
Unfiltered	Asia	Philippines	6	3	n < 5
Unfiltered	Asia	Singapore	5	3	n < 5
±1.5 SD	Asia	Philippines	5	3	n < 5
±1.5 SD	Asia	Singapore	4	3	n < 5
±1 SD	Europe	Czechia	4	9	n < 5
±1 SD	Asia	South Korea	14	4	n < 5
±1 SD	Asia	Philippines	5	2	n < 5
±1 SD	Asia	Singapore	4	2	n < 5

Table S6. Country-level sample sizes and hydrogen import cases under alternative filtering approaches, related to Table 2.

Region	Country	n Demand	n Supply	Median case	Mean case	High import	Low import
Unfiltered							
Europe	Germany	20	12	62	126	355	-128
Europe	Italy	13	14	112	41	277	-129
Europe	Netherlands	16	12	99	90	165	11
Europe	Belgium	13	10	125	114	156	69
Europe	Austria	9	7	29	15	51	14
Europe	Czechia	7	10	43	30	64	18
Asia	Japan	35	20	-124	-681	225	-515
Asia	South Korea	15	8	-28	35	29	-71
Asia	Thailand	13	6	-1	-19	65	-29
Asia	Indonesia	18	14	-65	-2,147	101	-721
Asia	Vietnam	8	11	-72	-64	100	-265
±1.5 SD							

Europe	Germany	18	10	33	71	198	-121
Europe	Italy	13	12	131	82	279	-53
Europe	Netherlands	14	11	95	80	139	1
Europe	Belgium	11	9	125	120	152	94
Europe	Austria	8	6	27	26	35	14
Europe	Czechia	7	9	43	40	64	18
Asia	Japan	32	18	-83	-104	216	-415
Asia	South Korea	14	8	-28	-24	25	-71
Asia	Thailand	12	5	4	18	64	-17
Asia	Indonesia	17	12	-30	-238	69	-426
Asia	Vietnam	7	9	-55	-38	15	-136
±1 SD							
Europe	Germany	15	7	40	63	151	-88
Europe	Italy	6	11	125	88	175	19
Europe	Netherlands	10	10	108	94	139	40
Europe	Belgium	7	9	125	138	152	125
Europe	Austria	7	6	29	29	40	17
Asia	Japan	31	18	-87	-117	210	-416
Asia	Thailand	11	5	1	11	39	-19
Asia	Indonesia	17	12	-30	-238	69	-426
Asia	Vietnam	7	9	-55	-38	15	-136

Table S7. Sectoral composition of global hydrogen demand in the GECO 2022 1.5 °C scenario in 2050 and derivation of the feedstock sensitivity, related to Figure 4 and Table 2.[S1]

Hydrogen use	Share of total H_2 demand
Transport, including H_2 input to e-fuels	78%
Iron and steel	12%
Combustion in industry and buildings	1%
Non-energy industrial feedstock, derived by difference	≈9%
Total	100%

Source: GECO 2022[S1], Section 4.1.2 and Figure 20; non-energy feedstock share calculated as 100% − 78% − 12% − 1% = 9%.

Methods S1. Sensitivity to non-energy hydrogen demand in JRC estimates, related to Figure 4 and Table 2.

Of the demand estimates retained in the main analysis, 28 are derived from the JRC Global Energy and Climate Outlook (GECO) series: 11 for Japan, 11 for South Korea and 6 for Indonesia. The values used in the meta-analysis correspond to hydrogen reported within total final energy consumption. They, therefore, include hydrogen demand in transport, buildings and industrial energy applications, including steelmaking, but exclude pure hydrogen classified as non-energy use, such as feedstock for ammonia production and refining. In the GECO modelling framework, hydrogen used as industrial feedstock is reported under "Non-energy uses", whereas hydrogen used in steelmaking is included in industrial energy demand.[S1]

To assess the relevance of the excluded feedstock component, we use the sectoral breakdown of global hydrogen demand reported in GECO 2022 for the 2050 1.5 °C scenario[S1]. Transport accounts for 78% of total hydrogen demand, including direct hydrogen use and hydrogen used for e-fuel production, while iron and steel account for 12%. Hydrogen combustion in industry and buildings contributes a further 1%. The remaining approximately 9% corresponds, by difference, to non-energy industrial feedstock use (Table S7).[S1] JRC identifies these uses primarily with petrochemical applications, including ammonia production and oil refining, and projects them to stabilise or decline towards 2050 as fertiliser use becomes more targeted and oil demand decreases[S1].

The approximately 9% feedstock share provides an indication of the magnitude of non-energy hydrogen use not represented in the JRC final-energy values used in the meta-analysis. We therefore apply a 10% feedstock add-back to all 28 JRC-derived demand observations as a sensitivity case. The adjustment is intended to test the influence of this system boundary on the meta-analysis rather than to replace the original literature values.

Following the 10% adjustment, Japan, South Korea and Indonesia remain net-surplus countries in the median, mean and low-import cases, leaving the corresponding aggregate estimates unchanged. The high-import case increases from 1,254.84 to 1,260.58 TWh, an increase of 5.74 TWh (0.46%). The literature-based import range therefore changes from 128–1,255 TWh in the main analysis to 128–1,261 TWh under the feedstock sensitivity.

Supplemental References

[S1] Keramidas, K. et al. Global Energy and Climate Outlook 2022: Energy trade in a decarbonised world. Joint Research Centre, European Commission (2022). https://doi.org/10.2760/863694
